# *Cis*-Antisense Transcription Gives Rise to Tunable Genetic Switch Behavior: A Mathematical Modeling Approach

**DOI:** 10.1371/journal.pone.0133873

**Published:** 2015-07-29

**Authors:** Antoni E. Bordoy, Anushree Chatterjee

**Affiliations:** 1 Department of Chemical and Biological Engineering, University of Colorado Boulder, Boulder, CO, United States of America; 2 BioFrontiers institute, University of Colorado Boulder, Boulder, CO, United States of America; Niels Bohr Institute, DENMARK

## Abstract

Antisense transcription has been extensively recognized as a regulatory mechanism for gene expression across all kingdoms of life. Despite the broad importance and extensive experimental determination of *cis*-antisense transcription, relatively little is known about its role in controlling cellular switching responses. Growing evidence suggests the presence of non-coding *cis*-antisense RNAs that regulate gene expression via antisense interaction. Recent studies also indicate the role of transcriptional interference in regulating expression of neighboring genes due to traffic of RNA polymerases from adjacent promoter regions. Previous models investigate these mechanisms independently, however, little is understood about how cells utilize coupling of these mechanisms in advantageous ways that could also be used to design novel synthetic genetic devices. Here, we present a mathematical modeling framework for antisense transcription that combines the effects of both transcriptional interference and *cis*-antisense regulation. We demonstrate the tunability of transcriptional interference through various parameters, and that coupling of transcriptional interference with *cis*-antisense RNA interaction gives rise to hypersensitive switches in expression of both antisense genes. When implementing additional positive and negative feed-back loops from proteins encoded by these genes, the system response acquires a bistable behavior. Our model shows that combining these multiple-levels of regulation allows fine-tuning of system parameters to give rise to a highly tunable output, ranging from a simple-first order response to biologically complex higher-order response such as tunable bistable switch. We identify important parameters affecting the cellular switch response in order to provide the design principles for tunable gene expression using antisense transcription. This presents an important insight into functional role of antisense transcription and its importance towards design of synthetic biological switches.

## Introduction

**Fig 1:**
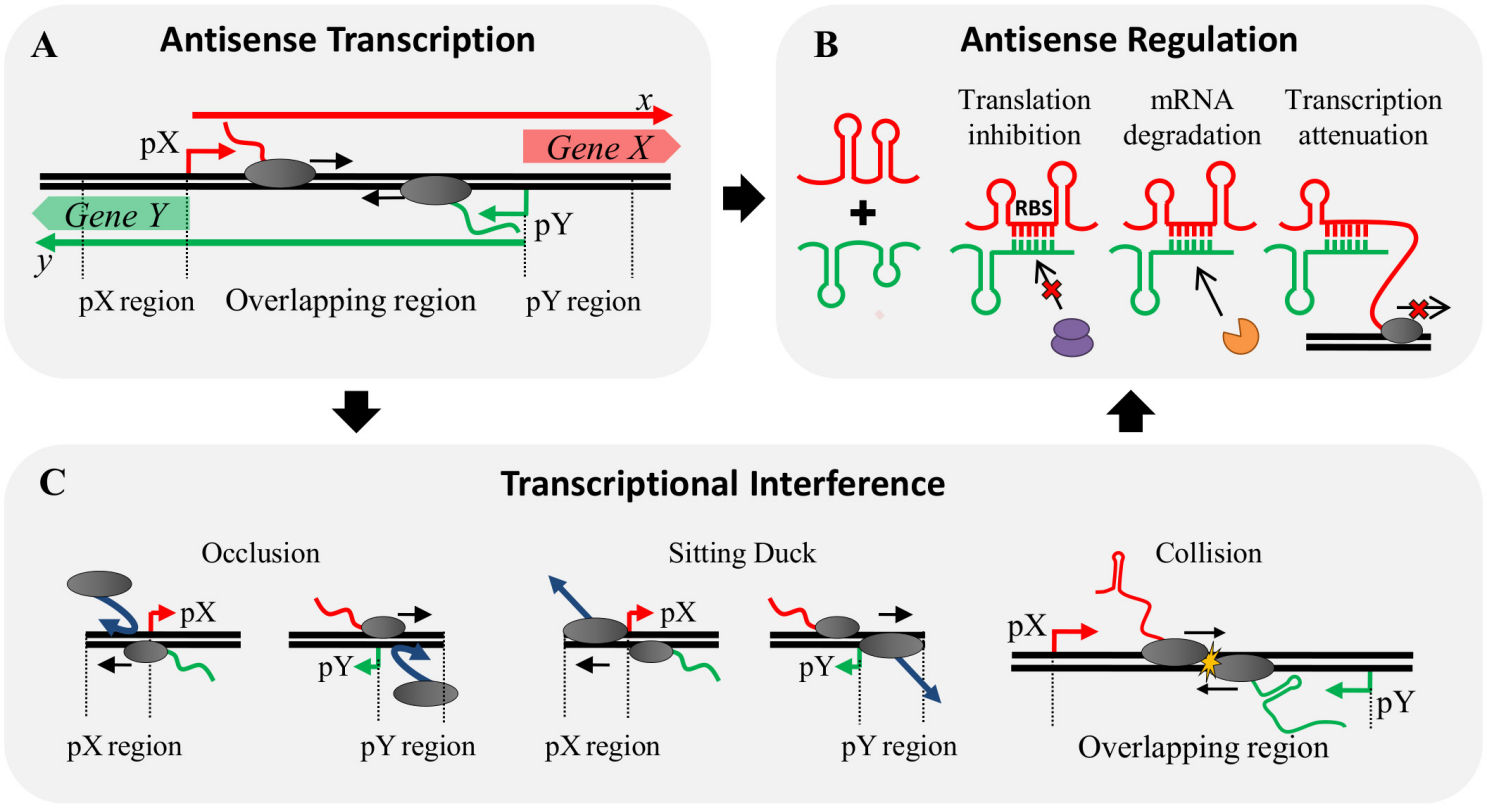
Antisense transcription mechanisms in a set of convergent genes. **(A)** General set of convergent promoters driving expression of genes *X* and *Y* synthesizing transcripts *x* and *y* (bold arrows), respectively. Such a system is susceptible to TI and produces overlapping transcripts that may participate in AR. **(B)** AR can cause translational inhibition, mRNA degradation and transcriptional attenuation due to the interactions that may exist between full-length sense and antisense transcripts as well as truncated RNA produced as a result of RNAP collisions, one of the reported TI mechanisms. **(C)** Mechanisms of TI: Occlusion caused by passage of an opposing elongating RNAP on the antisense promoter which hinders binding of RNAP to the sense promoter; Sitting duck interference, dislodgement of an initiation complex due to collision with an opposing elongating RNAP; and Collision between opposing elongating RNAP molecules that produces truncated RNA of different sizes susceptible to participate in AR. Both TI and AR mechanisms are likely to be coupled during antisense transcription.

Tunable regulation of gene expression is essential to overcome multitude of adverse environmental conditions encountered by biological systems [1,2]. Non-coding genomic regions and closely spaced promoters observed in genome-wide studies have revealed the role of transcription in controlling gene expression [3–7]. Often, when both DNA strands are transcribed in the same genomic locus, convergent arrangements of promoters are observed, leading to antisense transcription (Fig 1A). This phenomenon plays an important role in cell differentiation [8], stress responses [9,10], pathogenic processes [11,12], virulence [13], and development of life-threatening diseases [14–17]. Regulatory mechanisms linked with antisense transcription include *cis*-antisense RNA regulation (AR) and transcriptional interference (TI).

AR can arise between RNA transcripts encoded in opposite strands of the same DNA region, which are perfectly complementary, known as *cis-*antisense RNA interaction; or between transcripts encoded from different genome regions, which are only partially complementary, known as *trans-*antisense interaction. Antisense RNAs (asRNAs) are ubiquitous in all three domains of life and known to regulate gene expression in various ways [18–22]. asRNAs regulating sense mRNAs act via three predominant mechanisms (Fig 1B). Translation inhibition, whereby binding of the asRNA prevents translation of the mRNA by usually blocking its ribosomal binding site (RBS) [23] or, less commonly, other mRNA regions [24]. mRNA degradation, in which the resulting hybrid asRNA-mRNA double stranded RNA (dsRNA) is targeted for fast degradation by RNases (RNase III, E, etc.) [13,25–27]. Finally, transcriptional attenuation, where binding to the target mRNA disrupts its transcription [28–30]. Other less common mechanisms include degradation protection [11,31], inhibition of primer maturation and prevention of formation of an activator RNA pseudoknot [18,32].

During antisense transcription an additional mechanism affecting expression of neighboring genes is known as TI and is defined as the suppressive effect of transcription from one promoter on an adjacent promoter [33]. Presence of TI at various promoter arrangements has been shown to be ubiquitous [34–37]. For head to head, non-overlapping promoters, four TI mechanisms have been described (Fig 1C). Occlusion, whereby presence of a RNA polymerase (RNAP) originating from one promoter hinders the binding of a RNAP at the neighboring promoter [34,38]; sitting duck interference, in which an elongating RNAP collides with a promoter bound RNAP [36]; collision, in which two opposing elongating RNAPs collide while traversing the overlapping DNA, often producing truncated transcripts [39] and roadblock, where a DNA bound protein blocks movement of an elongating RNAP [40].

Despite the broad importance and extensive experimental determination of *cis-*antisense transcription, relatively little is known about its role in controlling cellular switching responses. Furthermore, majority of prior mathematical models have focused either on TI [41] or AR [42,43] mechanism alone, and little is understood about how cells utilize coupling of these mechanisms in advantageous ways that could also be used to design novel synthetic genetic devices. Here we present a discrete stochastic TI model that brings a new aspect to the previously existing TI model [41] as it calculates the rate of production of truncated RNA species produced as a result of RNAP collision during antisense transcription. The model predicts TI events occurring in a general set of convergent, non-overlapping promoters providing truncated and full-length RNA production rates, which are then used to solve a system of ordinary differential equations (ODEs) describing their presence. We show the tunability of TI through various parameters and that coupling of TI with AR gives rise to hypersensitive switches in expression of both antisense genes. In addition, we consider proteins encoded by the genes to regulate antisense transcription by implementing positive and negative feedback loops controlling the transcriptional process, and therefore affecting both TI and AR. Our approach reveals the regulatory potential that arises from coupling these three layers of gene regulation from convergent promoters. The model predicts multiple tunable responses, from first-order ramping switch to higher-order bistable switch by fine-tuning of the system parameters. Therefore, we identify the most important parameters affecting the cellular switch response in order to provide the design principles for tunable gene expression using antisense transcription. To the best of our knowledge, we present the first model for antisense transcription integrating three regulatory layers with the purpose of designing synthetic genetic devices taking advantage of antisense transcription.

## Methods

### Discrete TI model

#### RNAP binding, firing and elongation along the DNA

**Fig 2:**
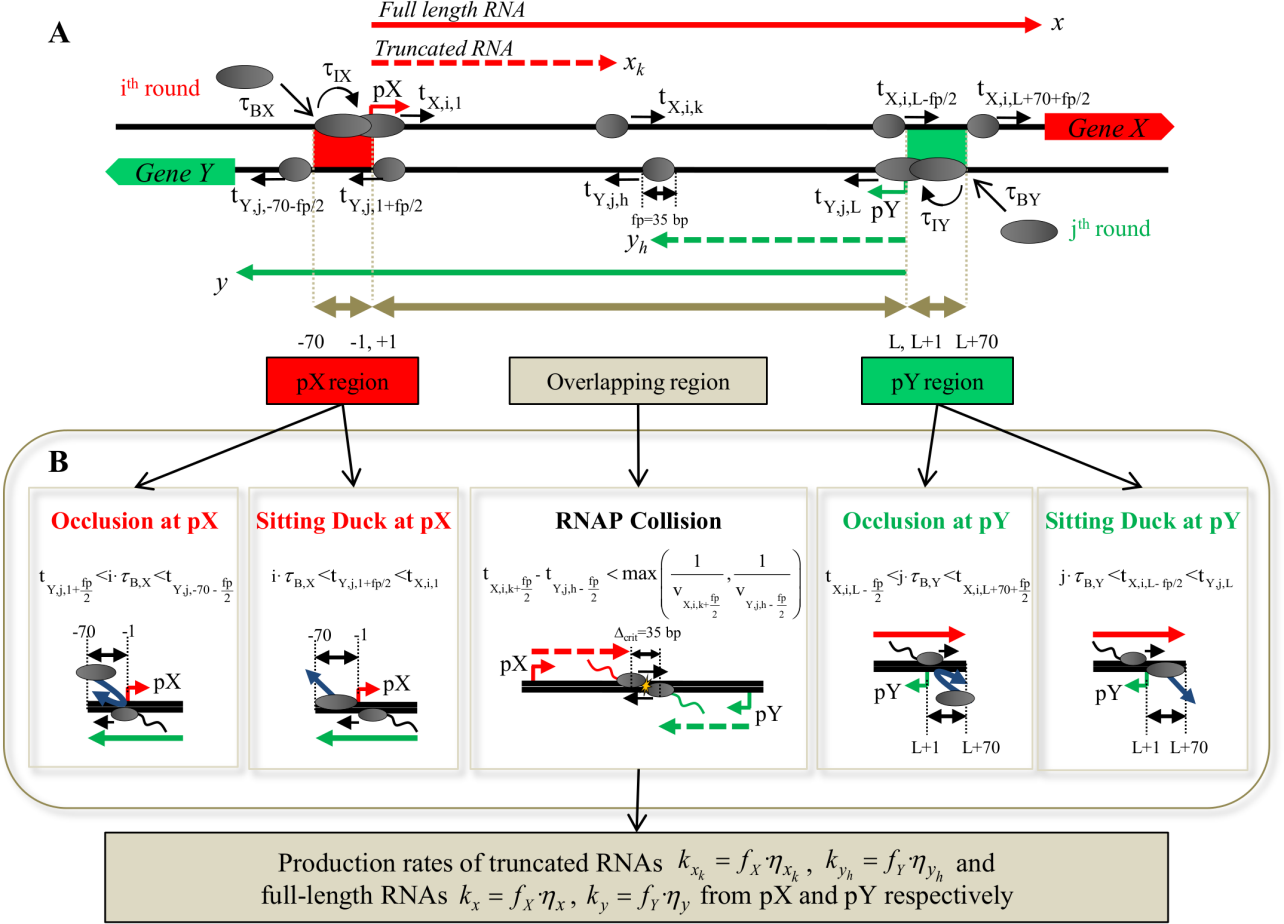
Algorithm for discrete TI model. **(A)** A pair of convergent promoters pX (present on sense/top DNA strand) and pY (present on antisense/bottom DNA strand) separated by overlapping DNA of length L is shown. Promoters pX and pY drive expression of genes *X* and *Y* respectively, which produce full-length transcripts *x* and *y* (denoted by bold arrows) respectively. For each i^th^ and j^th^ round of transcription from pX and pY promoters respectively, RNAP (denoted by large grey ovals) form DNA-bound RNAP complexes at the respective promoter region following a binding (τ_BX_ and τ_BY_) and initiation (τ_IX_ and τ_IY_) process. After firing, the center of RNAP moves to first position of the overlapping region to form an elongation complex (EC, denoted by smaller grey ovals). The time taken for each i^th^ EC (fired from pX) to reach k^th^ position on sense strand (t_X,i,k_) as well as the time taken for each j^th^ EC (fired from pY) to reach h^th^ position on the antisense (t_Y,j,h_) strand along the overlapping DNA are tracked. The footprint of an EC is denoted by *fp*. **(B)** For each i^th^ and j^th^ rounds of transcription, the model calculates the outcome of TI depending on the region where opposing RNAPs meet. Occlusion and sitting duck interference occur at the promoters pX (left panels) or pY (right panels), and RNAP collisions between ECs occur along the overlapping DNA (middle panel) following the mathematical constraints shown. Upon RNAP collision, one or both ECs on the sense and antisense strand fall off the DNA and result in production of truncated transcripts *x*_*k*_ and *y*_*h*_ (denoted by dashed arrows) from pX and pY respectively. In absence of any kind of TI, transcription is successful, producing a full-length transcript (*x*, *y*). Once 30,000 rounds of transcription from the stronger promoter have been calculated the net rate of production of full-length (*k*_*x*_ and *k*_*y*_) and truncated RNA ($k_{x_{k}}$ and $k_{y_{\text{h}}}$) are obtained.

We developed a discrete model that simulates RNAP traffic originating from a general set of two convergent, non-overlapping promoters pX and pY present on sense and antisense strands of DNA, driving the expression of genes *X* and *Y*, respectively. The model calculates the outcome of every single round of transcription by tracking binding and movement of RNAP along three different regions along the DNA: the pX promoter region from position -70 to -1, the intermediate overlapping region of length L, which spans between positions +1 and L, and promoter pY region from position L+1 to L+70 (Fig 2).

Transcriptional initiation follows the McClure model, which describes the multi-step transcription initiation process using two main steps [44–46]. Firstly, RNAP is considered to bind to promoters pX and pY at average binding times τ_BX_ and τ_BY_ respectively (Table 1) to form initiation complexes (IC). The IC then transitions into elongation complex (EC) after an average initiation time τ_IX_ and τ_IY_ at pX and pY promoters, respectively. During the initiation time the RNAP remains bound to the respective promoter while unwinding the double-stranded DNA (open complex). Promoter bound RNAP (IC) is assumed to occupy a footprint (fp) of 70 bp. Both RNAP binding (τ_BX_±0.05τ_BX_ and τ_BY_±0.05τ_BY_) and initiation times (τ_IX_±0.05τ_IX_ and τ_IY_±0.05τ_IY_) are assumed to follow a Gaussian distribution to account for inherent biological noise [47,48]. Average RNAP binding times lie in range of 5.5–55 s based on estimates for P_bla_ system in *E*. *coli* [49,50] and bacteriophage λ [51]. Average RNAP initiation time is assumed to be in a range of 5–16 s based on T7 RNAP [52]. We assume the time required for transition from IC to EC to be shorter than the binding time (τ_IX_<τ_BX_ and τ_IY_<τ_BY_), thus self-occlusion at both promoters is neglected [53]. Since τ_IX_<τ_BX_ and τ_IY_<τ_BY,_ time between two consecutive RNAP firing events coincides with the time between two consecutive RNAP binding events at the promoter. Therefore, the RNAP firing rates or frequencies *f*_X_ and *f*_Y_ from promoters pX and pY respectively are solely given by the inverse of the corresponding RNAP binding time (*f*_X_ = 1/τ_BX,_ *f*_Y_ = 1/τ_BY_). We define relative strength α of promoters pX-pY, as the ratio of RNAP firing rates (α = *f*_Y_/*f*_X_). α can vary between three states, α<1 when pX is more aggressive than pY promoter, α~1 when pX and pY have comparable strengths, and α>1 when pY is more aggressive than pX. RNAPs are assumed to be in excess, thus we consider no effect on transcription due to sequestering of transcriptional machinery [36]. The binding and firing events of RNAP at pX are assumed to be independent of binding and firing events at pY promoter and vice versa except for TI mechanisms.

**Table 1:** Parameters of discrete TI model.

Parameter	Description
τ_BX_	Average binding time at pX.
τ_BY_	Average binding time at pY.
*f*_X_	Firing rate at pX, defined as inverse of pX average binding time, *f*_X_ = 1/τ_BX_
*f*_Y_	Firing rate at pY, defined as inverse of pY average binding time, *f*_Y_ = 1/τ_BY_
τ_IX_	Average initiation time at pX
τ_IY_	Average initiation time at pY
t_X,i,k_	Time at which center of RNAP corresponding to the i^th^ round of transcription from pX reaches k^th^ position
t_Y,j,h_	Time at which center of RNAP corresponding to the j^th^ round of transcription from pY reaches h^th^ position
*η*_*x*_	Fraction of successful rounds of transcriptions relative to total rounds of transcription from pX
*η*_*y*_	Fraction of successful rounds of transcriptions relative to total rounds of transcription from pY
$\eta_{x_{k}}$	Fraction of rounds of transcription producing truncated RNA of size *k* relative to total rounds of transcription from pX
$\eta_{y_{h}}$	Fraction of rounds of transcription producing truncated RNA of size *h* relative to total rounds of transcription from pY
*k*_*x*_ = *f*_*x*_ *η*_*x*_	Transcription rate of full-length RNA *x* in presence of TI
*k*_*y*_ = *f*_*y*_ *η*_*y*_	Transcription rate of full-length RNA *y* in presence of TI
$k_{x_{k}}=f_{x}\eta_{x_{k}}$	Transcription rate of truncated RNA *x*_*k*_ (population of truncated RNAs longer than 60 bp)
$k_{y_{h}}=f_{y}\eta_{y_{h}}$	Transcription rate of truncated RNA *y*_*h*_ (population of truncated RNAs longer than 60 bp)

In case of successful RNAP firing, we assume elongation starts with the center of an EC RNAP discretely moving to the first position of overlapping DNA (+1 for pX, +L for pY). Footprint (fp) of an EC is considered to be 35 bp [54]. Movement of ECs is considered to occur in discrete steps [55] with an average velocity of 50 bp/s along the DNA following a Gaussian distribution (50±2.5 bp/s) based on experimental values [56]. The simulation calculates the time that RNAP center reaches each k^th^ and h^th^ position for every i^th^ and j^th^ round of transcription (t_X,i,k_, t_Y,j,h_) (t_promoter,round,position_) from pX and pY respectively. The time taken by the EC to cover this distance is given by the summation of product of RNAP velocity at a particular location along the DNA $\Delta t=\sum_{m=1}^{n}\frac{1}{v_{m}}$, where *n* is the number of nucleotides covered by the center of the EC, and *v*_*m*_ is the local velocity of the EC at the m^th^ position.

#### RNA production in absence of TI

In absence of TI, the RNAP molecules successfully transcribe and synthesize full-length RNAs *x* and *y* of length >L+70 (Table 2), i.e. length of the overlapping region plus length of the convergent promoter region (no further TI is considered beyond those positions). The rate of production of *x* and *y* is given by *k*_*x*_ = *f*_*X*_ and *k*_*y*_ = *f*_*Y*_, respectively. Only full-length RNAs *x* and *y* are considered to be accessible for translation into proteins, excluding translation of any kind of truncated RNA.

**Table 2:** List of species and corresponding models.

Species	Description	Models
*x*	Full-length RNA produced from promoter pX	TI, TI+AR, GN
*y*	Full-length RNA produced from promoter pY	TI, TI+AR, GN
*x*_k_	Truncated RNA produced from promoter pX	TI, TI+AR, GN
*y*_h_	Truncated RNA produced from promoter pY	TI, TI+AR GN
*x*:*y*	RNA hybrid between sense and antisense full-length RNA	TI+AR, GN
*x*_k_:*y*	RNA hybrid between sense truncated and antisense full-length RNA	TI+AR, GN
*x*:*y*_h_	RNA hybrid between full-length sense and antisense truncated RNA	TI+AR, GN
*x*_k_:*y*_h_	RNA hybrid between truncated sense and antisense truncated RNA	TI+AR, GN
X	Protein encoded by gene X	GN
Y	Protein encoded by gene Y	GN
Z	Activator protein	GN
W	Signaling molecule or upstream protein	GN
X:Z	Protein hybrid between X and Z proteins	GN
O_Y,T_	Total concentration of operator sites at promoter pY	GN
O_Y_	Unbound operator sites at promoter pY	GN

#### TI via occlusion

For j^th^ round of transcription, occlusion is considered to occur if EC of i^th^ round of transcription fired from pX traverses pY region when j^th^ RNAP tries to bind at pY (j·τ_BY_). Time taken to traverse an antisense promoter starts when front end of the EC reaches position L (EC center reaches position L-fp/2 at time t_X,i,L-fp/2_) and the back end of the EC leaves position L+70 (EC center leaves position L+70+fp/2 at time t_X,i,L+70+fp/2_). Upon occlusion (t_X,i,L-fp/2_<j·τ_BY_<t_X,i,L+70+fp/2_), j^th^ round of transcription from pY does not occur. Similarly, for i^th^ round of transcription at pX, occlusion is considered to occur if j^th^ EC fired from pY is traversing pX region (from -70 to -1) when i^th^ RNAP tries to bind at pX (t_Y,j,1+fp/2_<i∙τ_BX_<t_Y,j,-70-fp/2_), (Fig 2B).

#### TI via sitting duck interference (SDI)

SDI at pY is considered to occur when i^th^ EC fired from pX front end reaches the end of the overlapping region, i.e. position L, and encounters an IC stationed at pY (j∙τ_BY_<t_X,i,L-fp/2_<t_Y,j,L_), also known as sitting duck complex (SDC). SDI at pX occurs when j^th^ EC fired from pY front end reaches the end of the overlapping region, i.e. +1, and encounters an IC stationed at pX (i·τ_BX_<t_Y,j,1+fp/2_<t_X,i,1_), (Fig 2B). SDI is considered to have two different outcomes that impact transcription. SDCs are usually removed from the DNA upon collision with an opposing EC, in which case while the SDC causes no RNA to be produced, the EC continues transcribing. However, some SDCs are tightly bound to the DNA and can act as roadblocks [33,41]. Therefore, we assume that 10% of SDCs survive the collision with an EC, in which case the converging EC falls off the DNA producing a truncated RNA of length equal to the overlapping length minus half the RNAP footprint (L-fp/2).

#### TI via RNAP collision

If neither occlusion nor SDI occur, RNAP starts transcribing. However, ECs may still interfere with other opposing ECs or SDCs on the antisense promoter. RNAP collisions are considered to occur along the overlapping DNA (positions +1 to L) when two opposing ECs are separated by critical distance, Δ_*critical*_ or less. We consider the limiting case, where Δ_*critical*_ = RNAP footprint of an EC (35 bp). Δ_*critical*_ could be greater than the RNAP footprint when the torsional stress caused by simultaneous uncoiling of both DNA may cause the RNAPs to fall off the DNA [57]. Collision of two ECs is considered to result in one of two outcomes, either both ECs fall off the DNA strand, or only one EC falls off while the other continues to elongate (Fig 2B). We consider an RNAP survival probability of 80% in the case where one EC survives. We assume there is equal probability that it originated from pX or pY, resulting in an overall individual 40% probability to continue transcribing, as experimentally shown by Crampton *et al*. using Atomic Force Microscopy [57]. When one RNAP stops transcribing due to a collision event, it gives rise to a truncated RNA of length *x*_*k*_ or *y*_*h*_ depending on whether the EC originated from pX or pY respectively (Table 2).

### Model for TI based regulation (TI model)

The discrete TI model simulates 30,000 rounds of transcription for the stronger promoter and calculates the extent of each TI mechanism and resulting RNA production from each promoter. In order to calculate cellular RNA levels, we then consider a model consisting of four ODEs describing RNA mass balance in the cell accounting for their production, *k* (calculated by the discrete TI simulation), their degradation rate, λ, and dilution due to growth rate, μ (Equations 1–4, Table 3). The value of these parameters are based on experimental values obtained for *E*. *coli* [58,59] (S1 Table). In presence of TI, the net rate of production of full length RNA *x* and *y* is given by *k*_*x*_ = *f*_*X*_·*η*_*x*_, *k*_*y*_ = *f*_*Y*_·*η*_*y*_, where *η*_*x*_ and *η*_*y*_ are the fraction of successful rounds of transcriptions relative to total rounds of transcription from the respective promoter. Similarly, the net rate of production of truncated RNA *x*_*k*_ and *y*_h_ is given by $k_{x_{k}}=f_{X}\cdot \eta_{x_{k}}$, $k_{y_{h}}=f_{Y}\cdot \eta_{y_{h}}$, where $\eta_{x_{k}}$ and $\eta_{y_{h}}$ are the fraction of rounds of transcription producing a truncated RNA of a certain size relative to total rounds of transcription from the respective promoter (Table 1 and Fig 2B). It is important to note that in absence of TI all rounds of transcription produce full-length transcripts, and therefore *η*_*x*_ = 1 and *η*_*y*_ = 1 whereas no truncated RNAs are produced, i.e. $\eta_{x_{k}}=0$ and $\eta_{y_{h}}=0$. The steady state levels of full-length *x* and *y* transcripts and truncated transcripts were calculated by numerically solving equations 1–4 (Table 3) considering the initial species concentrations equal to zero.

**Table 3:** List of equations in the TI model, TI+AR model and gene network model.

Equation No.	Equation	Model
1	$\frac{\text{d}[x]}{\text{dt}}=k_{x}\text{-}\left(\mu +\lambda_{x}\right)[x]$	TI
2	$\frac{\text{d}[y]}{\text{dt}}=k_{y}\text{-}(\mu +\lambda_{y})[y]$	TI
3	$\frac{\text{d}[x_{\text{k}}]}{\text{dt}}=k_{x}{}_{{}_{\text{k}}}\text{-}(\mu +\lambda_{x_{\text{k}}})[x_{\text{k}}]$	TI
4	$\frac{\text{d}[y_{\text{h}}]}{\text{dt}}=k_{y}{}_{{}_{\text{h}}}\text{-}(\mu +\lambda_{y_{\text{h}}})[y_{\text{h}}]$	TI
5	$\frac{\text{d}[x]}{\text{dt}}=k_{x}{\text{-k}}_{\text{b}xy}[x][y{]\text{-k}}_{\text{b}xy_{\text{h}}}[x][y_{\text{h}}{]+\text{k}}_{\text{u}xy}\left(\left[x:y]+[x:y_{\text{h}}\right]\right)\text{-}\left(\mu +\lambda_{x}\right)[x]$	TI+AR GN
6	$\frac{\text{d}[y]}{\text{dt}}=k_{y}\;{\text{-k}}_{\text{b}xy}[y][x{]\;\text{-k}}_{\text{b}x_{\text{k}}y}[y][x_{\text{k}}{]+\text{k}}_{\text{u}xy}\left(\left[x:y]+[x_{\text{k}}:y\right]\right)\text{-}(\mu +\lambda_{y})[y]$	TI+AR GN
7	$\frac{\text{d}[x_{\text{k}}]}{\text{dt}}=k_{x_{\text{k}}}{\text{-k}}_{\text{b}x_{\text{k}}y}[x_{\text{k}}][y{]\text{-k}}_{\text{b}x_{\text{k}}y_{\text{h}}}[x_{\text{k}}][y_{\text{h}}{]+\text{k}}_{\text{u}xy}[x_{\text{k}}:y{]+\text{k}}_{\text{u}x_{\text{k}}y_{\text{h}}}[x_{\text{k}}:y_{\text{h}}]\text{-}(\mu +\lambda_{x_{\text{k}}})[x_{\text{k}}]$	TI+AR GN
8	$\frac{\text{d}[y_{\text{h}}]}{\text{dt}}=k_{y_{\text{h}}}{\text{-k}}_{\text{b}xy_{\text{h}}}[x][y_{\text{h}}{]\text{-k}}_{\text{b}x_{\text{k}}y_{\text{h}}}[x_{\text{k}}][y_{\text{h}}{]+\text{k}}_{\text{uxy}}[x:y_{\text{h}}{]+\text{k}}_{{\text{ux}}_{\text{k}}{\text{y}}_{\text{h}}}[x_{\text{k}}:y_{\text{h}}]\text{-}(\mu +\lambda_{y_{\text{h}}})[y_{\text{h}}]$	TI+AR GN
9	$\frac{\text{d}[x:y]}{\text{dt}}{=\text{k}}_{\text{b}xy}[x][y{]\;\text{-}(\text{k}}_{\text{u}xy}+\mu +\lambda_{x:y})[x:y]$	TI+AR GN
10	$\frac{\text{d}[x_{\text{k}}:y]}{\text{dt}}{=\text{k}}_{\text{b}x_{\text{k}}y}[x_{\text{k}}][y{]\;\text{-}(\text{k}}_{\text{u}xy}+\mu +\lambda_{x_{\text{k}}:y})[x_{\text{k}}:y]$	TI+AR GN
11	$\frac{\text{d}[x:y_{\text{h}}]}{\text{dt}}{=\text{k}}_{\text{b}xy_{\text{h}}}[x][y_{\text{h}}{]\;\text{-}(\text{k}}_{\text{u}xy}+\mu +\lambda_{x:y_{\text{h}}})[x:y_{\text{h}}]$	TI+AR GN
12	$\frac{\text{d}[x_{\text{k}}:y_{\text{h}}]}{\text{dt}}={\text{k}}_{\text{b}x_{\text{k}}y_{\text{h}}}[x_{\text{k}}][y_{\text{h}}{]\;\text{-}(\text{k}}_{\text{u}x_{\text{k}}y_{\text{h}}}+\mu +\lambda_{x_{\text{k}}:y_{\text{h}}})[x_{\text{k}}:y_{\text{h}}]$	TI+AR GN
13	$\frac{\text{d}[\text{X}]}{\text{dt}}{=\text{k}}_{\text{X}}[x{]\;\text{-k}}_{\text{XZ}}{[\text{X}][\text{Z}]+\text{k}}_{\text{uXZ}}[\text{X:Z}]\;\text{-}(\mu +\lambda_{\text{X}})[\text{X}]$	GN
14	$\frac{\text{d}[\text{Y}]}{\text{dt}}{=\text{k}}_{\text{Y}}[y]\text{-}(\mu +\lambda_{\text{Y}})[\text{Y}]$	GN
15	$\frac{\text{d}[\text{Z}]}{\text{dt}}{=\text{k}}_{\text{WZ}}{+\text{k}}_{\text{YZ}}{[\text{Y}]\;\text{-k}}_{\text{XZ}}{[\text{X}][\text{Z}]+\text{k}}_{\text{uXZ}}[\text{X:Z}]\text{-}(\mu +\lambda_{\text{Z}})[\text{Z}]$	GN
16	$\frac{\text{d}[\text{X:Z}]}{\text{dt}}{=\text{k}}_{\text{XZ}}{[\text{X}][\text{Z}]\;\text{-}(\text{k}}_{\text{uXZ}}+\mu +\lambda_{\text{XZ}})[\text{X:Z}]$	GN
17	$\frac{{[\text{O}}_{\text{Y}}]}{{[\text{O}}_{\text{Y,T}}]}=\frac{{\text{K}}_{\text{OY}}}{{\text{K}}_{\text{OY}}+[\text{X}]}$	GN
18	$f_{\text{Y}}=f_{\text{Y},\max}\frac{{[\text{O}}_{\text{Y}}]}{{[\text{O}}_{\text{Y,T}}]}+f_{\text{Y},\min}\left(1-\frac{{[\text{O}}_{\text{Y}}]}{{[\text{O}}_{\text{Y,T}}]}\right)$	GN

### Model for TI and Antisense RNA interaction based regulation (TI+AR model)

In addition to regulation of full-length RNA production in presence of TI, the TI+AR model accounts for the interaction between fully and partially complementary RNA species produced from the pX-pY locus. Four kinds of RNA interactions are considered, species *x*:*y* refers to RNA hybrid complex between full-length sense and antisense transcripts, *x*_*k*_:*y* and *x*:*y*_*h*_ represent truncated sense and full-length antisense RNA hybrid complexes and vice versa respectively; and *x*_*k*_:*y*_*h*_ represents complex formed between truncated sense and truncated antisense RNA (Table 2). Based on lengths of naturally occurring *cis-*antisense RNAs known to participate in AR, we assume that the entire population of truncated RNAs longer than 60 bp would be able to fold into stem loops, hairpins and bulges required to catalyze interactions [60]. Mass balance analysis leads to a set of eight ODE equations (Equations 5–12 in Table 3) considering the rate of production of transcripts *x*, *y*, *x*_*k*_ and *y*_*h*_ provided by the discrete TI model, *k*, their mutual RNA interactions, k, degradation rates, λ, and dilution due to volume expansion caused by cell growth, μ. All RNA duplexes are assumed to be unavailable for translation and rapidly targeted for degradation [60]. Since all interacting RNAs are considered to have the requisite secondary structure to participate in RNA interactions, the binding of the four RNA complexes are assumed follow a second order kinetics with rate constants (k_b*xy*_, ${\text{k}}_{\text{b}}{}_{x_{\text{k}}y}$, ${\text{k}}_{\text{b}xy_{{}_{\text{h}}}}$, and ${\text{k}}_{\text{b}x_{\text{k}}y_{{}_{\text{h}}}}$) within the range of 10^6^−10^7^ M^-1^ s^-1^, based on experimentally determined values in literature (Equations 9–12 Table 3, S1 Table) [61,62]. We assume species *x*:*y*, *x*_*k*_:*y* and *x*:*y*_*h*_ to have a first order unbinding kinetics with the same unbinding rate constant, k_uxy_. Since truncated RNA hybrid complex *x*_*k*_:*y*_*h*_ contain shorter regions of complementary, we consider a higher unbinding rate constant, ${\text{k}}_{{\text{ux}}_{\text{k}}{\text{y}}_{\text{h}}}$ (S1 Table). The steady state levels of full-length *x* and *y* transcripts and truncated transcripts were calculated by numerically solving equations 5–12 (Table 3). The initial concentrations for all species were considered to be zero.

### Gene Network Model (GN model)

Based on the architecture of naturally occurring *cis*-antisense systems, we developed a representative *cis*-antisense transcription based gene regulatory network where one of the gene encodes for a transcriptional factor or an inducer molecule that regulates the expression of the antisense gene [39,63–66]. In order to describe the presence and effect of proteins, TI+AR model was extended to include six additional equations (Equations 13–18) to account for the production and interaction of protein species. We consider genes X and Y encode proteins X and Y respectively which are functionally related. Proteins X and Y are produced by translation of only full-length RNA *x* and *y* respectively following first order kinetics based on previously described naturally occurring systems [63,67,68] (Equations 13–14 and S1 Table). Protein X implements a negative feedback loop acting as a transcriptional repressor which binds to the operator site of pY promoter (O_Y_) regulating its strength (Table 1). Upon binding of X, pY is repressed, increasing its RNAP binding time, therefore decreasing its firing rate (*f*_Y_). Assuming binding of repressor X to operator site O_Y_ reaches rapid equilibrium, the fraction of unbound operator sites is given by Equation 17. The RNAP binding rate at pY is considered to be proportional to the concentration of unbound operator sites at pY as described by Equation 18, where *f*_Y,min_ and *f*_Y,max_ are the RNAP firing rates at pY under repressed and de-repressed conditions, respectively. Protein Y implements a positive feedback by indirectly activating production of a transcriptional activator Z, which is considered to bind protein X and relieve repression at pY following a second order kinetics (Equations 15–16). Protein Z may also function as a signaling molecule produced independent of Y activation. We consider Z is also produced from an external source, W, which could be a host gene, a gene from an acquired plasmid, or an environmental signal (released by other cells), following a zero order kinetics, k_WZ,_ (Equation 15). Dissociation of X:Z complex is considered to follow first order kinetics (Equation 16). Parameter nomenclature and their numerical values used are summarized in S1 Table.

For every value of k_WZ_, the steady state levels of cellular species in presence of TI, AR and protein feedback were calculated by numerically solving equations 5–18 (Table 2) in two steps. Since the RNAP firing rate of the inducible promoter pY depends on protein X levels, the first step involved calculating an initial guess for X (Equation 17–18). To achieve this, an initial guess for transcriptional rates for *x* RNA, and truncated RNA *x*_k_ and *y*_h_ was set at *k*_*x*_ = 0.05 nM/s and $k_{x}{}_{{}_{\text{k}}}$ = $k_{y}{}_{{}_{\text{h}}}$ = 0.01 nM/s respectively. This initial guess was used to obtain steady state X levels by solving Equations 5–18. The updated protein X level was used to calculate the transcriptional rates for each RNA species using the discrete TI model. Finally, the system of equations (Equations 5–18) was solved a second time with the updated values of transcriptional rates to obtain steady state levels of all cellular species.

#### Software

Discrete TI model was developed in Matlab. Equations used in TI and TI+AR models were solved using the differential equation solver ode45 in Matlab. GN model equations were solved using Mathematica. Parameters values used were either obtained from the literature or estimated within the biological range and are summarized in S1 Table [49–52,58,59,61,62,67–69]. Only physiologically achievable solutions were considered.

#### Hill coefficient analysis

Response curves were fitted to the form $\frac{k_{\max}\alpha^{H}}{{\text{K}}^{H}+\alpha^{H}}$ where *H* is the Hill coefficient representing the sharpness of the switch response, *k*_*max*_ represents the maximum value of the response, K denotes the value of α that results in half of the maximum value; α being the relative promoter strength. Fitting was performed using Method of Least Squares in Microsoft Excel. Adjusted R^2^ values were >0.95 for all fits. For fits of all *x* switches, *k*_*max*_ = 10.3±0.3 (avg±SD) and K = 0.8±0.1. For fits of all *y* switches, *k*_*max*_ = 39.0±7.8 and K = 1.7±0.2. Positive Hill coefficient values indicate increase in RNA production with increase in α whereas, negative values indicate decrease in RNA production with α increase.

## Results

### Tunable biological switch response using TI alone

**Fig 3:**
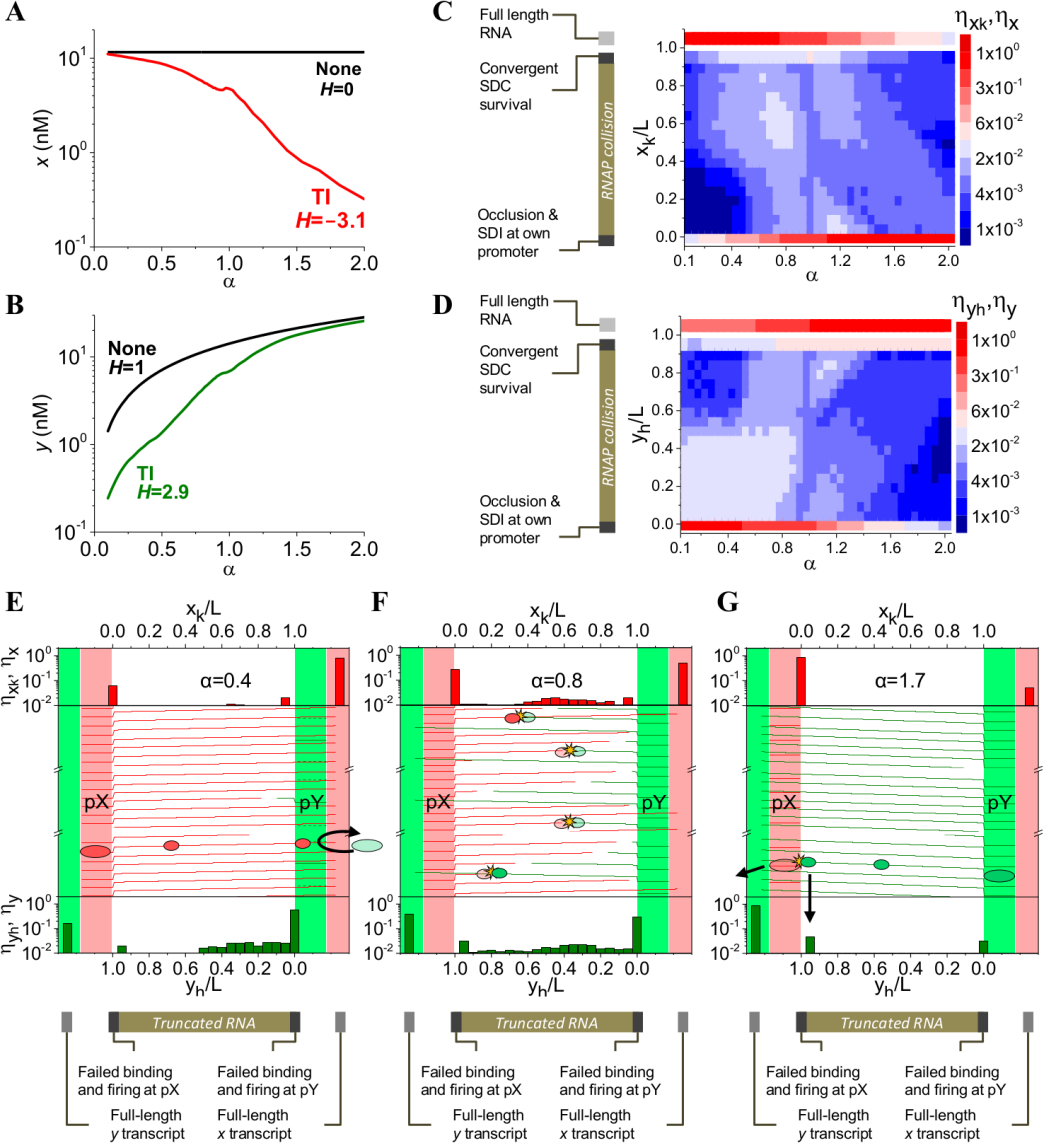
TI gives rise to a switch-like response. **(A, B)** Reciprocal switch in expression of full-length *x* transcript level (A) and full-length *y* transcript level (B). *x* and *y* expression is inversely correlated, while *x* levels decrease as α increases, *y* levels increase. *H* denotes the value of Hill coefficient (see Methods) **(C, D)** Expression maps for full-length (*η*_*x*_, *η*_*y*_) and truncated ($\eta_{x_{k}},\;\eta_{y_{h}}$) transcripts from pX (C) and pY (D) respectively. Fraction of rounds of transcription which failed to fire (due to occlusion or sitting duck interference, SDI) are depicted at positions *x*_k_/L = 0 and *y*_h_/L = 0. Production of truncated RNA transcripts due to survival of SDCs at pY and pX promoter is represented at positions *x*_k_/L = 0.95 and *y*_h_/L = 0.95, respectively. Fraction of successful full-length transcripts (*η*_*x*_, *η*_*y*_) appears at positions *x*_k_/L>1 and *y*_h_/L>1. **(E)** Mechanistic representation of occlusion at pY for α = 0.4. Middle panel shows representative trajectories of ECs along the DNA when occlusion at pY is the dominant mechanism. Horizontal green dashed lined at pY region (middle panel) indicate rounds of transcription that were occluded. Top and bottom panels show transcriptional fraction of full-length (*η*_*x*_, *η*_*y*_) and truncated RNA transcripts ($\eta_{x_{k}},\;\eta_{y_{h}}$) originated at pX (top panel) and pY (bottom panel), respectively. Fraction of rounds of transcription which failed to fire (due to occlusion or sitting duck interference) are depicted at positions *x*_k_/L = 0 (top panel) and *y*_h_/L = 0 (bottom panel)**. (F, G)** Analogous representations showing RNAP collision for α = 0.8 (F) and SDI at pX for α = 1.7 (G) as the dominant mechanism, respectively. For **E, F**, and **G** panels, opaque ellipses represent RNAPs that continued transcribing after TI occurred while translucent ellipses denote RNAPs that were not able to bind or remain bound to DNA. The simulations shown in panels A-G correspond to L = 400 bp, τ_BX_ = 20 s, τ_IX_ = 12 s and τ_IY_ = 9.5 s, and α values varied from 0.1 to 2 by varying τ_BY_ from 2 to 40 s. Initiation time always remained smaller than binding times in order to avoid self-occlusion.

Biological conditions can cause the relative strengths of pX and pY promoters, α, to change from α<1 to α>1, giving rise to a switch response in gene expression (see Methods). For the chosen system of convergent promoters with a given length of overlapping DNA between them (here L = 400 bp, S1 Table), we obtained the production of truncated and full-length transcripts over a range of α values between 0.1 and 2 (Fig 3A and 3B). We assume pX is a constitutive promoter with fixed average RNAP binding and initiation times (τ_BX_ = 20 s, τ_IX_ = 12 s), whereas pY is an inducible promoter with a variable RNAP binding time (τ_BY_ = ατ_BX_), but a fixed RNAP initiation time (τ_IY_ = 9.5 s). These parameter values are used hereafter unless otherwise stated. The condition tested here assumes EC’s do not pause and move with an average velocity of 50 bp/s (⟨v_x_⟩ = ⟨v_y_⟩ = 50 bp/s) from pX and pY promoters.

The steady state levels of full-length *x* and *y* transcripts in presence of TI alone (Equations 1–4, Table 3) show that TI can give rise to a considerably sharper switching response compared to the no TI case (Fig 3A and 3B). In presence of TI, the constitutive pX promoter behaves as an inducible promoter with steady state full-length *x* levels decreasing nearly 2 orders of magnitude as α increases from 0.1 to 2. Conversely, full-length *y* RNA also increases nearly 2 orders of magnitude, thus demonstrating a reciprocal switch response. This is an outcome of a 41-fold decrease in *η*_*x*_ and a 5-fold increase in *η*_*y*_ (see Methods, Table 1) as α changes from 0.1 to 2 (Fig 3C and 3D). The sharpness of the switching response is characterized by higher Hill coefficient of -3.1 for *x* and 2.9 for *y*; compared to Hill coefficient of 0 for *x* and 1 for *y* in absence of TI. Similar switches in gene expression due to TI have been observed in naturally occurring systems such as the 30-fold decrease in expression of the *gal* promoter [70] and the 5.6-fold repression of bacteriophage 186 pL promoter in the convergent pR-pL system [36].

Changing α can also result in one or more TI mechanisms (occlusion, SDI and RNAP collision) to play a dominant role that can allow for tuning of full-length transcript expression as well as production of specific truncated RNA distributions. Carefully controlling TI may provide an opportunity to maximize production of specific sizes of truncated transcripts that may have the potential to interact with the antisense RNA [39,63,71]. For example, changing α from 0.1 to 0.75 can cause rate of production of truncated RNA of length 260 bp (at position x_k_/L = 0.65) to increase by 10.7-fold due to increased RNAP collisions (Fig 3C). As a general trend, collisions occur closer to the weaker promoter, i.e. close to pY for α<1 and move towards pX promoter as α increases. When pX is the aggressive promoter, such that α<1, most transcripts from pX end up as a full-length RNA (Fig 3C) whereas those from pY result in occlusion or short truncated RNAs (Fig 3D). The opposite happens when pY is the aggressive promoter, such that α>1 (Fig 3C and 3D).

When α is far from 1 (one promoter is much stronger than the neighboring promoter), occlusion is the dominant TI mechanism since high frequency of ECs severely hinders binding of RNAP at the weaker promoter. This is evident in the range of low α values from α = 0.1 to α = 0.4 (Fig 3C and 3D), where majority of the rounds transcription from the stronger pX promoter occlude RNAP binding at pY (Fig 3E). This is consistent with naturally occurring systems where occlusion has been proposed to be the dominant TI mechanism due to high values of relative promoter strength. For example, α = 0.03 has been previously reported for the P_gal_ promoter relative to the P_L_ promoter [70]. Upon occlusion gene expression levels of full-length RNA (x_k_/L>1) are higher from the stronger pX promoter, whereas minimal from the weaker pY promoter (Fig 3E). Although stochasticity allows few binding events to occur at pY, majority of RNAPs that escape occlusion undergo RNAP collision shortly after elongation starts due to high density of opposing ECs originating from the stronger promoter (Fig 3E, bottom panel).

For α closer to 1, RNAP collision and SDI at both promoters gain importance (Fig 3C and 3D). For instance, at α = 0.8, RNAP collision becomes the dominant mechanism (Fig 3F). Since we assume RNAP binding and initiation times, and velocity profiles to follow Gaussian distributions in order to account for inherent biological noise, collisions occur at several loci along the overlapping DNA region ranging from 0 to L-fp/2. Such distributions are affected by the probability of RNAP survival (S1 Fig). As expected, an increase in L leads to increased collision caused due to higher RNAP residence time (S2 Fig). Changing α to 1.7 causes SDI at pX to become the dominant TI mechanism. Under this condition high levels of full-length *y* transcript are expressed in addition to a narrow truncated RNA size distribution, mainly corresponding to *y*_h_ transcripts of length L-fp/2 due to SDC survival (Fig 3G). Thus, maximization of SDI can be used to increase production of truncated RNA with length similar to the length of the overlapping region that may further participate in AR. SDI at pY is maximum at α = 1, with an associated transcriptional fraction $\eta_{x_{k}}$ = 0.032 (at L-fp/2 or *x*_k_/L = 0.95) that then decreases by 18.5-fold at α = 2 (Fig 3C and 3D). As α increases, SDI is less important at pY while still being present at pX (Fig 3C and 3D). The frequency of SDI is also strongly influenced by RNAP initiation time at pX and pY. As τ_IX_ increases, SDI at pX gains importance and RNAP collision rate decreases, leading to slight sharpening of the switch response (S3A–S3C Fig). Analogous effect in *y* switch is seen when τ_IY_ is increased, while no effect is observed for *x* switch (S3D–S3F Fig).

### RNAP pausing biases switching response

In addition to changing relative RNAP firing rates, RNA production can be affected by the presence of pause sites. The effect of RNAP collision can be enhanced by increasing the residence time of RNAP in the overlapping region; such as during a pausing event, the local speed of RNAP is reduced, resulting in an increased RNAP residence time at a certain base pair

**Fig 4:**
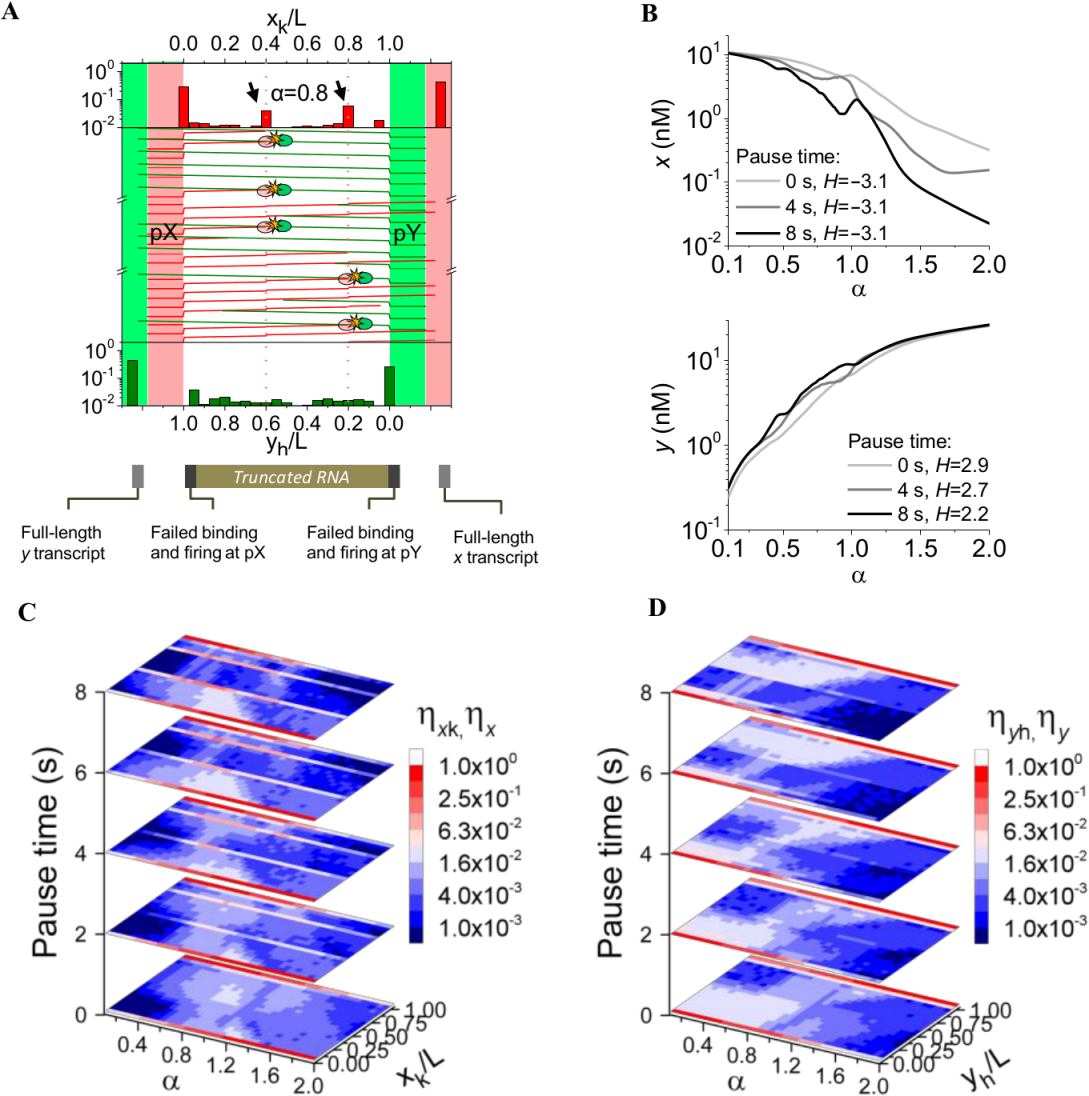
TI in the presence of RNAP pause sites. **(A)** Mechanistic representation of RNAP collision in presence of two pause sites in the sense strand at positions 160 bp (x_k_/L = 0.4) and 320 bp (x_k_/L = 0.8). Pause times are 2 s. Increased production of truncated RNA at the pause sites due to enhanced probability of collision is indicated by arrows. **(B)** Switch response in *x* levels maintains sharpness as pause time increases but the dynamic range of *x* is widened more than one order of magnitude. *H* denotes the value of Hill coefficient (see Methods). **(C)** Expression maps for truncated and full-length RNA from pX at different pause times. Truncated RNA production increases as pause time increases. **(D)** Expression maps for truncated and full-length RNA from pY at different pause times.

Previous experimental data indicate that transcriptional pausing is ubiquitous in natural systems and usually occurs at every 100–200 bp for durations of 1–6 s on an average [72]. To evaluate the contribution of RNAP pausing to TI we assume RNAP pausing along the sense strand of the DNA at positions 160 bp (x_k_/L = 0.4) and 320 bp (x_k_/L = 0.8). As a result the average velocity of ECs in the sense strand decrease ⟨v_x_⟩<50 bp/s, whereas ⟨v_y_⟩ = 50 bp/s. Paused RNAP are treated similar to SDCs initiating at the promoter region, i.e. in a collision event between an EC and a paused RNAP, we consider that the latter will fall off the DNA in 90% of the cases while in the remaining 10% of the cases the ECs will stop transcribing. Interestingly, the broad range of sizes of truncated RNA from pX in absence of RNAP pausing converges to narrower distributions in presence of 2 s long pauses [72] due to increased probability of head-on collision that arise from the longer residence time of RNAP at the pause site. The truncated RNA sizes expectedly correspond well with specific location of pause sites (Fig 4A). As an example, when 2 s pauses are considered, at α = 0.8 (where collision is the dominant mechanism in absence of pauses, Fig 3F), production of *x*_k_ truncated RNA at pause site 160 bp (x_k_/L = 0.4) and 360 bp (x_k_/L = 0.8) increase 3.6- and 4.3-fold, respectively. Thus, pausing can be used as design tool to bias production of a truncated RNA predominantly along just one DNA strand. Importantly, such a strategy can be used to design synthetic constructs that express specific truncated RNA products including riboswitches or non-coding RNAs participating in RNA interaction.

Pause sites not only increase the frequency of RNAP collision but also can decrease the transcription rate of full length RNA, and thus impact the switch response. Presence of longer pauses (4 and 8 s) at the same pause sites on sense strand did not significantly impact the sharpness of the *x* switch (*H* = -3.1), however, it caused an increase in the dynamic range of *x* (between α = 0.1 and 2) from 35-fold for no pause (0 s), to 70-fold for 4 s pause, and 475-fold for 8 s pause. Conversely, for *y* switch the dynamic range (*y* at α = 0.1/ *y* at α = 2) was slightly reduced from 106-fold for no pause (0 s), to 84-fold for both 4 s and 8 s pauses (Fig 4B). Pauses also cause shifts in the truncated RNA distribution originating from pY. Both effects become more important as the pause time increases (Fig 4C and 4D). In absence of pauses, short transcripts from pY are produced whereas at long pause times majority of truncated RNAs from pY are longer than 200 bp and therefore more susceptible to AR. Finally, it is important to note that experimental values of RNAP pause sites measured *in vivo* in *E*. *coli* show that pause durations can last more than 20 s, which would enormously increase the chances of RNAP collision [51]. The possibility of further engineering synthetic constructs by modifying the production of truncated RNA that are able to undergo AR expands the range of regulation during antisense transcription.

### Coupled effect of TI and *cis-*antisense RNA interaction during convergent transcription gives rise to higher-order response

**Fig 5:**
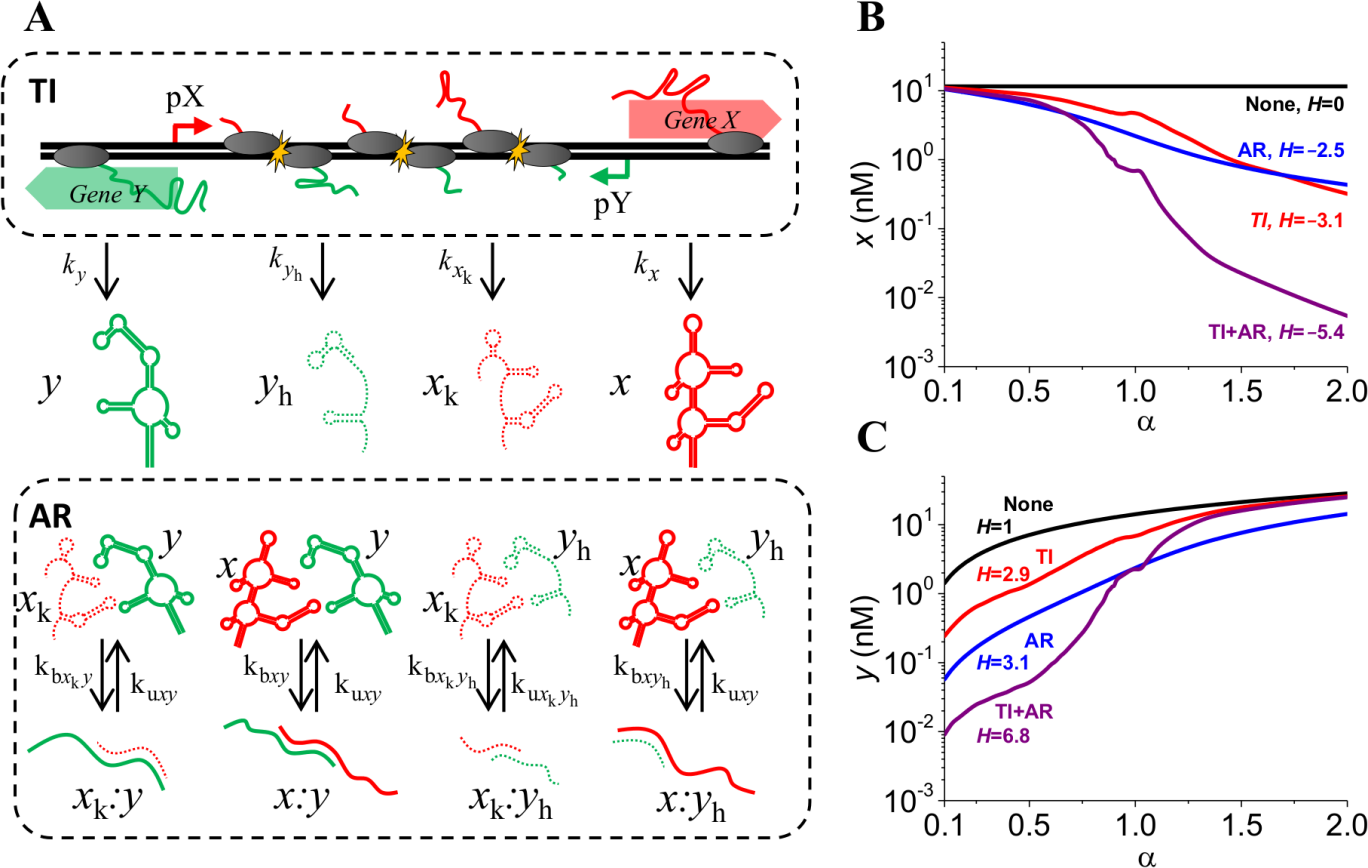
Effect of TI and AR on switch response. **(A)** TI model predicts the rate of production of truncated (dashed) and full-length RNA (bold). Interactions between different species arise due to presence of secondary structures and complementary sequences. RNA duplexes are targeted for fast degradation. **(B, C)** Different switch responses of full-length *x* and *y* levels depending on presence or absence of TI and AR. Sharpest response is obtained when both mechanisms are coupled, *H* denotes value of Hill coefficient.

We postulate that both full-length RNA and a fraction of the truncated RNA may play a role in regulating expression of antisense gene through silencing of the antisense RNA. A model based on the proposed mechanism of TI and AR during antisense transcription is shown in Fig 5A. The underlying hypothesis is that RNA interaction between complementary sense and antisense truncated as well as full-length RNA species allows sharpening of the switch response compared to TI or AR mechanism alone. Using a two part mathematical model we first estimate the rate of generation of full-length RNA species (*k*_*x*_ and *k*_*y*_) and truncated RNA species ($k_{x_{k}}$ and $k_{y_{h}}$) using the discrete TI model, and then use these transcription rates to solve equations 5–12 (Fig 2, Methods section).

We analyzed the four potential combinations of mechanisms that occur during convergent transcription: presence of both TI and AR regulatory mechanisms (TI+AR), exclusively TI (TI), exclusively AR (AR), and none of the mechanisms of TI and AR (None). We eliminated TI by setting the transcription rate of full-length *x* and *y* RNA to the firing rate *f*_X_ at pX and *f*_Y_ at pY, respectively (Methods section). To eliminate AR, we set the rate constant for RNA interactions in corresponding equations to zero. Again we varied α from α<1 to α>1 by modifying τ_BY_ while keeping τ_IY_, τ_BX_ and τ_IX_ constant. While the expression level of *x* stayed constant when varying α for the “None” case, *x* levels changed for AR, TI and TI+AR cases (Fig 5B and 5C), despite pX being a constitutive promoter. As α increases, strength of pY becomes greater than pX, and therefore gene expression of *y* increases. Interestingly, the sharpest hypersensitive response for both *x* and *y* (Hill coefficient, *H* = -5.4 for *x* and 6.8 for *y*, Methods section) was observed in the TI+AR case, followed by AR and TI cases, which had similar sharpness (TI: *H* = -3.1 for *x* and 2.9 for *y*; AR: -2.5 for *x* and 3.1 for *y*). If none of the mechanisms were considered the Hill coefficient was 0 for *x* as expected because constitutive pX strength is independent of α and 1 for *y* (No effect of α, *y* depends solely on *f*_Y_). It is important to note that hypersensitive switches confer the system with a higher-order response compared to a first-order response characterized by a linear or ramping behavior. As α increases from 0.1 to 2, TI+AR case allows three orders of magnitude change in *x* levels whereas no change is observed for “None” case. Similarly, *y* levels for TI+AR case vary three orders of magnitude compared to just one order for “None” case. The switch observed in *x* and y levels from α = 0.1 to α = 2 is approximately an order of magnitude wider in the TI+AR case when compared to TI only and AR only cases. For the chosen parameters, switch in gene expression is very similar for TI only and AR only cases. It is important to note that the switching response depends on RNA binding, unbinding and degradation rates of the interacting species.

### Protein regulation creates bistable switch behavior

**Fig 6:**
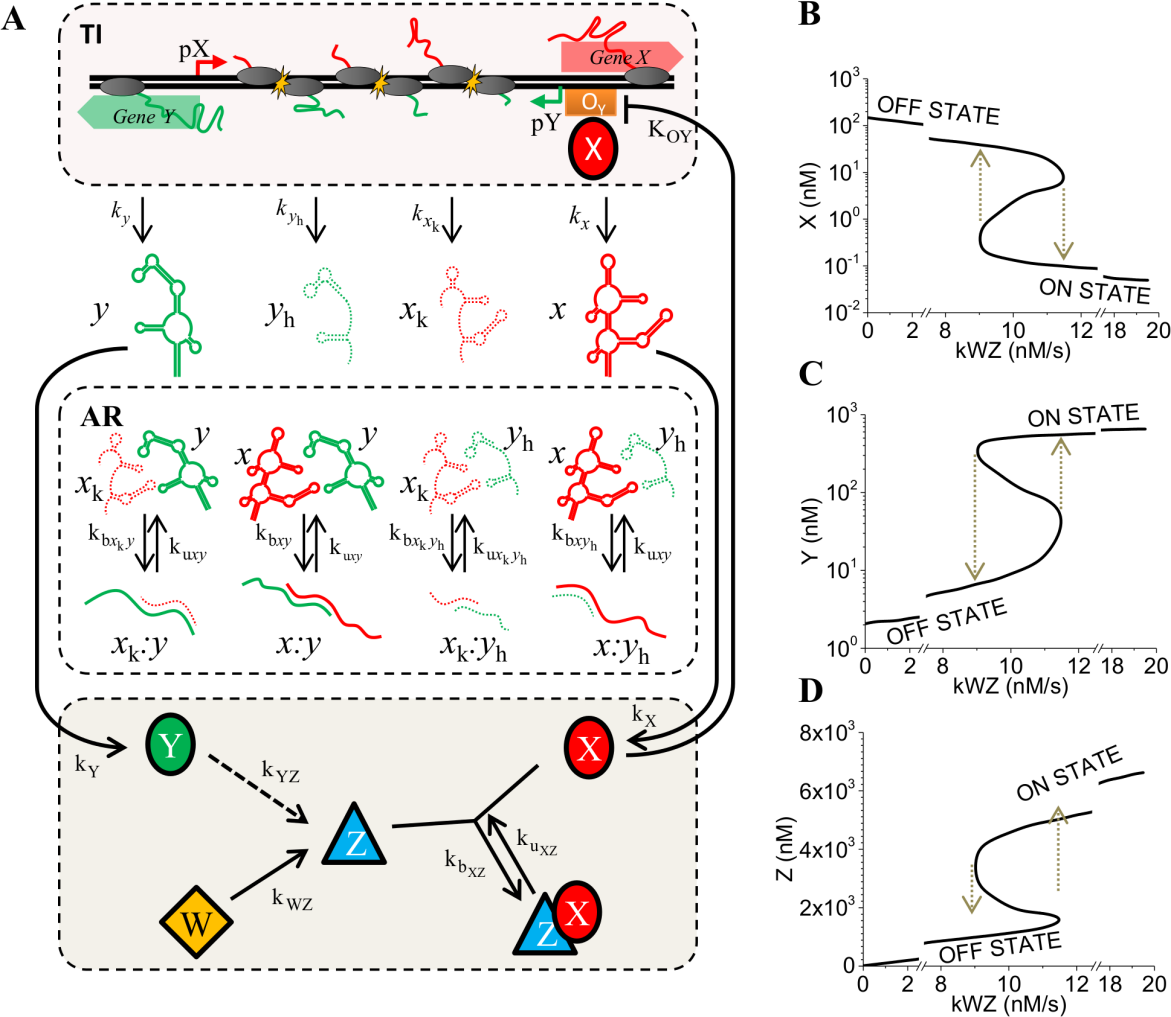
Antisense transcription coupled with protein feedback gives rise to bistability. **(A)** Successful full-length *x* and *y* transcripts that do not undergo antisense interaction are free to be translated into proteins X and Y respectively. Production of inducer molecule, Z, is indirectly activated by protein Y. Protein X implements a negative feed-back loop by binding to operator site O_Y_ and repressing promoter pY. Z binds to X and relieves the repression of pY promoter. **(B-D)** These multiple regulatory layers enable cells to demonstrate a higher-order a bistable switch response to different rates of production of W (denoted by k_WZ_). ON state is characterized by production of proteins Y and Z while OFF state is characterized by production of protein X. Threshold k_WZ_ value to switch between OFF to ON states is 11.5 nM/s whereas the threshold for the inverse switch between ON to OFF states occurs at k_WZ_ = 8.8 nM/s.

We next consider regulation of the transcriptional process when protein products X and Y, encoded by genes *x* and *y* respectively, exert negative and positive feedback to create a gene regulatory network based on *cis-*antisense transcription (see Methods). We consider that protein X implements a negative feed-back loop by controlling pY strength. Protein Y in turn acts as an antagonist of protein X, and through the indirect activation of protein Z production implements a positive feed-back loop by relieving pY repression via binding of Z to X (Fig 6A). In this and following section, α only varies from α = *f*_Y,min_/*f*_X_ = 0.37 to α = *f*_Y,max_/*f*_X_ = 1.74, corresponding to the repressed state and de-repressed state of pY, respectively (S1 Table). We numerically solved equations 5–18 to analyze the steady state behavior of the system in response to different rates of production (k_WZ_) of transcriptional activator/signaling molecule Z. Steady state solutions show that coupling TI and AR with feedback from regulatory proteins give rise to a characteristic bistable switch response. When bistability is present, cells can adopt two experimentally observable distinct states depending on the production rate of Z (Fig 6B) [73]. The bistable curve comprises the characteristic S-shaped section where multiple steady states reside. Two of these are stable steady states that correspond to ON (lower curve, Fig 6B) and OFF state of the switch (upper curve, Fig 6B), whereas the unstable steady state in the middle is not observed experimentally. At low rates of production of Z the systems exists in an OFF state which is characterized by low expression levels of Y and Z (Fig 6C and 6D), but high expression levels of repressor X (Fig 6B). As k_WZ_ increases the system continues to be in the OFF state until it reaches a threshold rate of 11.5 nM/s, where the systems transitions to the ON state characterized by nearly two orders of magnitude increase in Z and Y levels and decrease in repressor X level. Conversely, once the switch is turned ON, the system continues to be in the ON state until the production rate k_WZ_ drops below 8.8 nM/s. The system thus demonstrates a sophisticated hysteresis response to Z production rate, marked by well separated ON to OFF states occurring at different threshold rates. It is important to note that both hypersensitive and bistable switches are considered higher-order because their non-linearity indicates cooperation between different mechanisms integrating the system. In the model proposed here interactions arise between the three regulatory layers: TI, AR and protein regulation.

### Tunability of bistable switch behavior

We next performed a sensitivity analysis to evaluate the impact of individual parameters on the steady state response in order to identify those that allow fine tuning of the dose-response curve. Parameters were divided into three groups: (i) transcriptional parameters, including the ones that affect the TI model and therefore influence the RNAP collision loci and truncated RNA distribution (from *f*_X_ to L in S1 Table), (ii) RNA species parameters, including binding and unbinding constants between the different hybrids and their respective degradation rates (from k_bxy_ to $\lambda_{x_{\text{k}}y_{\text{h}}}$ in S1 Table) and (iii) protein species parameters, including translational rates, binding and unbinding of protein molecules and their respective degradations (from k_X_ to K_OY_ in S1 Table). While keeping the values of the remaining parameters constant, each parameter of the second and third groups was decreased and increased 2-fold. However, each transcriptional parameter was modified in a smaller range so that it remained in the same range of values used in previous sections. Interestingly, parameter tuning led to different dose-response curves ranging from ramp-like behavior to a bistable switch. In this section, we describe some of the parameters that the bistable response curve was most sensitive to; parameters that did not significantly modify the response are only briefly mentioned. The former can be used to tune the cellular response of synthetic genetic constructs based on *cis-*antisense transcription.

**Fig 7:**
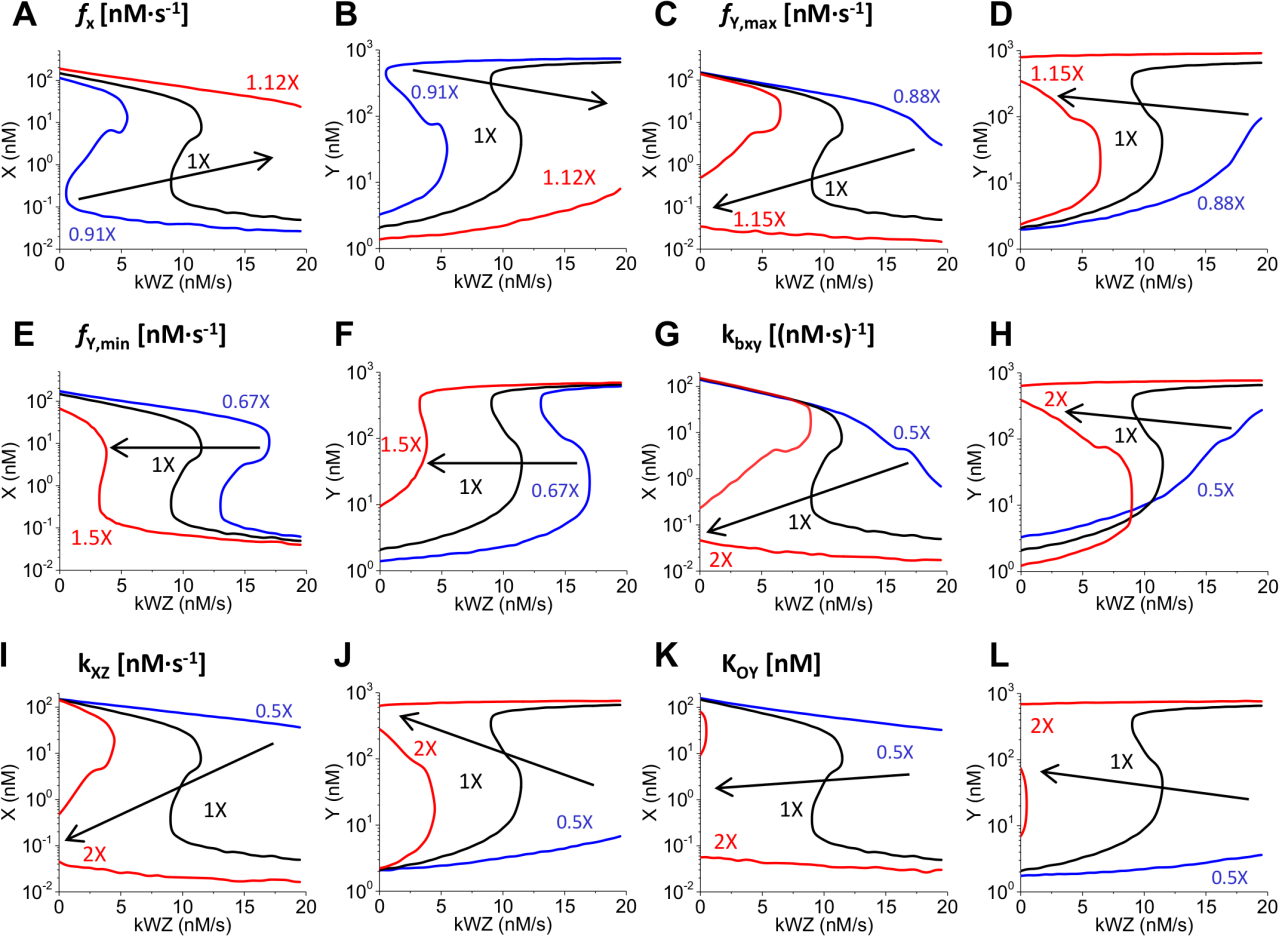
Parameters in GN model that impact bistable switch behavior. Examples of some of the parameters that had a greater effect when fine-tuned. Protein X and Y levels are shown for each modified parameter. Arrows indicate the way the curve shifts as the following parameters are individually tuned relative to the values shown in S1 Table. **(A, B)** *f*_X_, firing rate at pX. **(C, D)** *f*_Y,max_, firing rate at pX at de-repressed state. **(E, F)** *f*_Y,min_, firing rate at pX at repressed state. **(G, H)** k_bxy_, binding rate of full-length transcripts *x* and *y*. **(I, J)** k_XZ_, binding rate of protein complex X:Z. **(K, L)** K_OY,_ equilibrium binding constant of protein X at O_Y_ site.

In general, fine tuning of the parameters related with relative promoter strength α modified both the bistable range and type of switch behavior. Bistable region is highly sensitive to *f*_X_ as a 12% increase (or 1.12 times the values shown in S1 Table, also denoted as 1.12X) caused the bistable switch to shift to higher values of k_WZ_, with eventual loss of bistability and transition into a ramping switch response (Fig 7A and 7B). Decreasing *f*_X_ by 9% (or 0.91X) caused the bistable curve to shift to lower values of k_WZ_ needed to observe the bistable region. Further decrease caused the bistable switch to become irreversible. It is also important to note that relative promoter strength at the de-repressed state of pY (α = *f*_Y,max_/*f*_X_) strongly influences the shape of the dose-response curve (Fig 7C and 7D). As *f*_Y,max_ increases, the de-repressed pY becomes stronger and the bistable region shifts to lower values of k_WZ_ because less full-length *x* transcripts are obtained, whereas more full-length *y* transcripts are synthesized due to TI, at the same rates of production of Z. This results in lower levels of protein X and therefore repression at O_Y_ operator site is more easily relieved. At *f*_Y,max_ lower than 0.93X, which corresponds to weaker strength of de-repressed pY promoter compared to pX, we observe the bistable switch to transition into an ultrasensitive response. Further decrease in *f*_Y,max_ yields to a Michaelian behavior (*f*_Y,max_ = 0.88X). As pY at the repressed state gets stronger (*f*_Y,min_ increases), bistable range shrinks, at the same time the curve is shifted to lower k_WZ_ values. For *f*_Y,min_ = 1.5X we observe bistability for a k_WZ_ range ~0.6 nM/s (Fig 7E and 7F). On the other hand, higher initiation times at the antisense promoter increases the levels of full-length sense transcript whereas decreasing levels of truncated sense transcripts. However these changes did not modify the bistable range. Longer overlapping regions increased production of truncated RNA levels and resulted in higher hybrid RNA species, however changing L did not significantly modify the bistable range.

Interestingly, *x* and *y* binding kinetics also had a significant effect on the system (Fig 7G and 7H). As k_bxy_ increases, binding of full-length RNA transcripts increases full-length transcript sequestration. This caused widening of the bistable range, indicating that higher antisense interaction rates can serve as a noise filter due to increase in the separation between the thresholds to switch from ON to OFF. For low k_bxy_ values (0.5X) a Michaelian response is observed, where X levels decrease over 2 orders of magnitude as k_WZ_ increases from 0 to 20 nM/s. A 4-fold increase in k_bxy_ (2X) transforms the response into a bistable switch, with X levels corresponding to OFF and ON states separated by 4 orders of magnitude, along with bistable region of nearly 10 k_WZ_ units. Loss of bistability as k_bxy_ decreases suggests that AR is a key regulatory player in order to obtain a bistable switch. This was confirmed by studying the previous None, TI, AR and TI+AR cases in presence of the protein regulation, and observing loss of bistability for None and TI cases (S4 Fig). ${\text{k}}_{{\text{bx}}_{\text{k}}\text{y}}$ and ${\text{k}}_{{\text{bxy}}_{\text{h}}}$ slightly shifted the bistable curve to higher and lower values of k_WZ_, respectively.

Degradation rate of full-length *x* transcript significantly impacted the system. As expected, both *x* and X levels at k_WZ_ = 0 nM/s were decreased 6.6- and 7-fold, respectively when *λ*_*x*_ increased 4-fold, which also led to a 61.5-fold narrower bistable region at lower k_WZ_ values (S5A and S5B Fig). In the case of *λ*_*y*_, it also had a notorious effect on the bistable range (S5C and S5D Fig). A 26% increase in the value of *λ*_*y*_, caused loss of bistability. On the other hand, as *λ*_*y*_ decreases, the higher accumulation of full-length *y* transcript causes a sharp increase in the levels of inducer, Z, that binds to repressor X and results in a wider range of k_WZ_ values where pY can compete with pX, thereby expanding the bistable region. $\lambda_{x_{k}}$ and $\lambda_{y_{h}}$ slightly shifted the curve to the left and the right respectively; similar changes were caused by ${\text{k}}_{{\text{b}}_{x_{k}y}}$ and ${\text{k}}_{{\text{b}}_{xy_{h}}}$. Degradation rates $\lambda_{x_{k}y}$, $\lambda_{xy_{h}}$ and $\lambda_{x_{k}y_{h}}$ did not have a significant effect on the bistable curve although, as expected, these modified the levels of their respective hybrid RNA species.

Regarding the third group of parameters involving proteins, we observed that all of them significantly influenced the shape of the dose-response curve. Increased translational rate of protein X shifted the bistable region to higher values of k_WZ_, and as expected, also led to higher levels of protein X at the OFF state. Increasing translation rate of protein Y expanded the bistable region. Translational rate of protein Y, k_Y_, greatly impacts both protein Y and Z levels, as Z is indirectly activated by Y. Change in degradation rates of proteins X and Y had an opposite effect to changes in translational rates. Another important parameter is the binding kinetics of repressor and inducer molecule, k_XZ_, which also affects the robustness of the switch-like behavior by expanding the bistable region when increased (Fig 7I and 7J). Increases in k_XZ_ values greater than 72% led to an irreversible bistable switch, whereas bistability was lost turning to an ultrasensitive response when k_XZ_ was decreased below 43% of its original value. Importantly, the system is also sensitive to K_OY_, the parameter controlling the binding of repressor to operator site O_Y_, making it an important fine-tuning element (Fig 7K and 7L). Higher K_OY_ values implies weaker repression of pY; in turn a stronger pY competes more effectively with pX, thus lower levels of transcripts *x* are produced, resulting in lower k_WZ_ values required to turn on expression of protein Y.

## Discussion

Convergent arrangements of promoters are widespread in archea, bacteria and eukaryotes [18,74,75]. Although prevalent in shorter compact genomes in prokaryotes, the presence of thousands of such antisense gene pairs in relatively larger eukaryotic genomes [3,76,77] indicates that antisense transcription is an important regulatory mechanism conserved over evolution [78]. Our mathematical model highlights the regulatory advantage offered by coupled effects of TI and AR based regulation during antisense transcription. Using TI alone cells can achieve a hypersensitive switching response (Fig 3A–3D). We show that TI exerted by an inducible promoter on a convergent constitutive promoter, can cause the latter to demonstrate inducible expression (Fig 3A and 3B). Depending on the relative strengths of the promoters, different mechanisms of TI may dominate. Our model shows that RNAP collision has greater effect when promoters have similar strength (α close to 1). This is evident from previous studies on P_Q_-P_X_ convergent promoters of pCF10 plasmid in *E*. *faecalis* [39,71], under repressed conditions when both promoters have similar strengths the constitutive P_X_ promoter exerts nearly 10 fold decrease in activity of the P_Q_ promoter. Occlusion and SDI occur more frequently when one promoter is much more aggressive (α far from 1). This is exemplified by the pL-pR system of convergent promoters in coliphage 186, where, as a result of SDCs, a 5.6-fold reduction in activity of the weaker lysogenic (pL) promoter was observed, resulting in switching between the lysogenic and lytic states [36]. Experiments [36,39,51] also indicate that minor differences in strengths of convergent promoters can give rise to significant TI, such as during convergent transcription in the P_R_-P_RE_ promoter pair of bacteriophage λ where TI from the weaker P_RE_ promoter resulted in a 5.5 fold decrease in expression from the stronger P_R_ promoter due to occlusion [51].

Previously, Sneppen *et al*. developed stochastic, numerical (mean field) and analytical approaches to fit TI *in vivo* data on sets of convergent promoters [41]; but concluded that the analytical model turns increasingly inaccurate as promoter strengths become similar or when the overlapping distance is large, in both cases overestimating interference at the weak promoter. Even though our approach is different, both models agree that: (1) SDI increases as the initiation time approaches the binding time; (2) occlusion becomes more important at the weaker promoter as the strength of the stronger promoter increases, i.e. α<<1 and α>>1, and (3) TI increases as the overlapping region becomes longer due to larger number of collisions. Therefore both models are in agreement, with the advantage of being able to calculate production of truncated RNAs in our model.

A less explored aspect of TI relates to the potential regulatory role of cis antisense RNA produced from both convergent promoters. In nature, both short [13,79] and long antisense RNAs [13,80–82] have been shown to participate in antisense interaction in various bacterial and mammalian systems. Similar to our model, previous antisense RNA models reported by Levine *et al*. [42] and Mehta *et*. *al* [43] also used a second order RNA binding kinetics to describe RNA interaction between sense and antisense RNA transcripts. These models only accounted for RNA interaction between transcripts expressed *in trans*, which is analogous to the AR only model shown in Fig 5. The cellular concentrations and the switching response of RNA predicted by our AR model corroborates with that reported by Levine *et al* and Mehta *et al*, thus highlighting the generality of our model. Our model further shows that adding *cis*-RNA interaction along with TI mechanism can considerably sharpen the switching response with as high as 3 orders of magnitude difference in of full-length RNA levels (Fig 5B and 5C). The relative stoichiometry of sense and antisense transcripts influences the final extent of suppression, which is in turn influenced by the generation rate and half-life of the transcripts. We also demonstrate situations where production of specific sizes of truncated RNA can be enhanced for AR, such as in the presence of pause sites (Fig 4).

Only few experimental systems have accounted for the role of truncated RNA including the *prgQ/prgX* operon of pCF10 plasmid in *E*. *faecalis* [39]. In this system truncated *cis*-antisense RNA transcripts with sizes ranging between 100–200 nt and 80–200 nt were expressed from convergent promoters P_Q_ and P_X_ respectively, and were more prominent when the promoters were of similar strengths, than when P_Q_ activity was 10 times stronger than P_X_. Furthermore, these truncated transcripts were shown to inhibit expression of both full-length *prgX* and *prgQ* mRNA expression. Similarly, the *ubiG/mccBA* operon of *Clostridium acetobutylicum* has been shown to produce truncated RNA of various sizes ranging between 200–700 nt [83]. These truncated RNA lack Rho-dependent terminator structures at 3’ end, and their expression has been shown to be independent of RNase III and RNAse J1/J2 cleavage, potentially indicating that the termination mechanism could be based on RNAP collision.

Antisense transcription can result in complex cellular behavior especially in context of a biological gene network. Presence of a feedback loop, implemented in our generalized system by the control of promoter pY strength by protein X encoded from the convergent pX promoter enables the system to behave in a bistable fashion (Figs 6 and 7A–7L). Such gene networks with feedback loops have been observed in naturally occurring systems where sRNA plays an important role. OmrA and OmrB form a direct feedback regulation of OmpR, as well as RybB and σE transcription factor [84]. Indirect feedback regulation also exists as in the RyhB regulation of Fur [84]. We show that, in presence of a protein regulatory network, coupled effect of TI and AR is capable of demonstrating ramping, Michaelian (hyperbolic), ultrasensitive, as well as reversible and irreversible bistable behaviors (Fig 7A–7L). The bistable response is more sensitive to certain a set of parameters (such as *f*_X_, *f*_Y,max,_ *f*_Y,min,_ $k_{x_{\text{k}}}$, $k_{y_{\text{h}}}$, k_bxy_, K_OY_); which highlights the opportunity to tweak cellular responses when designing synthetic genetic devices based on *cis-*antisense transcription. Even for the parameters the system is more sensitive to, majority of them can be modified up to 4-fold without losing bistability. This confers the system with robustness as the noisy fluctuations are not expected to cause changes greater than 10% or 0.1-fold as previously shown in Elowitz *et al*. [85].

Reversible bistable switches are especially important for their robustness, allowing cells to make important decisions to adapt to new environments such production of antibiotics [63], plasmid conjugation [86], motility [87] or virulence status [88]. Some of these decisions involve the whole population and are usually require cellular communication via quorum sensing by means of a signaling molecule [89]. Bistability reduces the influence of signal noise that is inherent to biological systems due to stochasticity in cellular processes and environmental conditions. It does so by conferring the system with separation of threshold values in the production rate of signaling molecule to switch from one state to the other. The *prgQ/prgX* operon of *E*. *faecalis* [28,39] controlling conjugative transfer of drug resistance between cells has been shown to demonstrate bistable switch response using antisense transcription. On the other hand, the CopR system of *B*. *subtilis* controlling plasmid copy number has been speculated to be regulated by the three regulatory layers presented here: TI, AR and protein regulation of the transcriptional process, but no bistability has been observed [64]. In *ubiG/mccBA* operon of *C*. *acetobutlyicum*, both mechanisms of TI and RNA interaction confer a genetic switch regulating the expression of *ubiG* operon, which contains genes required for conversion of methionine to cysteine [83]. Similarly, antisense transcription has also been shown to facilitate two distinct bistable phenotypes in infectious pathogen *Bordetella* [90]. Finally, antisense transcription was recently shown to be important for functioning of circadian clocks [91].

Recent discovery of many new small RNAs, often with unknown function, indicate the presence of promoters opposite to protein-coding regions [92–94]. Most of these promoters in antisense orientation may express *cis*-antisense RNA not discovered yet that regulate the protein encoded by the antisense gene. Better understanding of TI and AR will provide important insights into biological functioning of microorganisms as well as higher organisms and development of improved biological systems. Given that promoter strength, overlapping DNA sequence and length is highly tunable, antisense transcription could be exploited to tweak naturally existing networks or create novel networks for synthetic biology applications. Moreover, genetic engineering taking advantage of these layers of regulation could allow us to develop engineered cells acting as biological sensors that perform different functions depending on the environmental conditions. Finally, although widespread presence of antisense transcription exists, further experimental characterization studies need to be done to understand whether antisense transcription is a fortuitous phenomenon in in nature or a well-designed set-up for gene regulation that could be controlled even at the genome scale.

## Supporting Information

S1 Table List of parameters TI model, TI/AR model and Gene Network Model. (DOCX)

S1 FigEffect of RNAP survival upon collision on RNA distribution.
**(A, B)** Expression maps of full-length (*η*_*x*_, *η*_*y*_) and truncated ($\eta_{x_{k}},\;\eta_{y_{h}}$) transcripts originating from pX (A) and pY (B), respectively for a range of percentage of RNAP survival in event of RNAP collision. As RNAP survival increases less truncated RNA is produced (bluer central region). **(C)** Switch response in full-length *x* and *y* transcript levels becomes sharper for lower percentage of RNAP survival, at 80% the Hill coefficient is -3.1 for *x* and 2.9 for *y*, at 40% it is -4.0 for *x* and 4.4 for *y*, and at 0% it is -7.8 for *x* and 8.2 for *y*.(TIFF)

S2 Fig Effect of various initiation time times on RNA distribution. **(A, B)** Effect of τ_IX_. Maps of truncated and full-length transcripts originating from pX (A) and pY (B), respectively, at different initiation intervals at pX, τ_IX_. Longer initiation times at pX increase probability of sitting duck interference at pX. Dislodgement of more sitting duck complexes at pX cause decreased RNAP collision and lowers expression of full-length *x* transcripts. **(C)** The switch response of *x* and *y* RNA at different τ_IX_ values. *x* transition becomes sharper at higher τ_IX_ values. **(D, E)** Effect of τ_IY_. As the initiation time at pY increases, analogous effect is seen. Sitting duck interference at pY increases and less RNAP collisions occur. **(F)** Switch response in *x* and *y* levels at different τ_IY_ values. *y* levels are lowered at higher τ_IY_.
(TIFF)

S3 Fig Effect of overlapping length on RNA size distribution. **(A, B)** Expression maps of truncated and full-length transcripts originating from pX (A) and pY (B), respectively, at different values of overlapping length, L. Longer overlapping regions yield to increased RNAP collisions. **(C)** Enhanced collisions lower the expression of full-length *x* and *y* transcripts. In this case, no clear trend was observed for the Hill coefficient.
(TIFF)

S4 Fig Antisense Regulation is required for bistable behavior. **(A, B)** Levels of full-length *x* (A) and *y* (B) transcripts, respectively, for None, TI, AR and TI+AR cases of antisense transcription in presence of the gene regulatory network implemented by proteins X, Y and Z. Bistability is only observed when AR is present (AR and TI+AR cases). When TI is coupled with AR (TI+AR case) the bistable range is expanded and the switch response is widened.
(TIFF)

S5 Fig Effect of full-length RNA degradation on bistability. Protein X and Y levels are shown for each modified parameter. Arrows indicate the way the curve shifts as the tuned parameter increases. **(A, B)** *λ*_*x*_, degradation rate of full-length *x*. **(C, D)** *λ*_*y*_, degradation rate of full-length *y*.
(TIFF)
