# Supplementary material for: Cis-Antisense Transcription Gives Rise to Tunable Genetic Switch Behavior: A Mathematical Modeling Approach
Source: PLoS One. 2015 Jul 29;10(7):e0133873. doi: 10.1371/journal.pone.0133873 (PMC4519249; doi:10.1371/journal.pone.0133873)
Supplement: S1 Table — (DOCX) [file pone.0133873.s001.docx]

S1 Table. List of parameters TI model, TI/AR model and Gene Network Model

| Parameter | Description | Estimated value  for bistability | Remarks/ References | Units | |
| --- | --- | --- | --- | --- | --- |
| *f*_x_ | Firing rate at pX | 5.00×10^-2^ | [49-51] | nM·s^-1^ | |
| *f*_Y,max_ | Firing rate at pY in de-repressed state | 8.695×10^-2^ | [49-51] | nM·s^-1^ | |
| *f*_Y,min_ | Firing rate at pY in repressed state | 1.85×10^-2^ | [49-51] | nM·s^-1^ | |
| $\text{τ}_{\text{IX}}$ | Average initiation time at pX | 12 | [52] | s | |
| $\text{τ}_{\text{IY}}$ | Average initiation time at pY | 9.5 | [52] | s | |
| L | Overlapping region length | 400 | Greater than promoter  bound distance (70 bp) | bp | |
| $k_{x_{\text{k}}}$ | Production of *x*_k_ truncated RNA | 1.0×10^-2^ | Based on TI simulations | nM·s^-1^ | |
| $k_{y_{\text{h}}}$ | Production of *y*_h_ truncated RNA | 1.0×10^-2^ | Based on TI simulations | nM·s^-1^ | |
| $\text{k}_{\text{bxy}}$ | Binding rate *x*:*y* | 8.0×10^-3^ | [61, 62] | (nM·s)^-1^ | |
| $\text{k}_{\text{uxy}}$ | Unbinding rate *x*:*y*, *x*_k_:*y* , *x*:*y*_h_ | 3.0×10^-4^ | [61, 62]. Slower than *x*:*y* binding rate | s^-1^ | |
| $\text{k}_{\text{bx}_{\text{k}}\text{y}}$ | Binding rate *x*_k_ :*y* | 1.1×10^-2^ | [61, 62] | (nM·s)^-1^ | |
| $\text{k}_{\text{bxy}_{\text{h}}}$ | Binding rate *x*:*y*_h_ | 1.1×10^-2^ | [61, 62] | (nM·s)^-1^ | |
| $\text{k}_{\text{bx}_{\text{k}}\text{y}_{\text{h}}}$ | Binding rate *x*_k_:*y*_h_ | 1.7×10^-2^ | [61, 62] | (nM·s)^-1^ | |
| $\text{k}_{\text{ux}_{\text{k}}\text{y}_{\text{h}}}$ | Unbinding rate *x*_k_:*y*_h_ | 1.5×10^-3^ | [61, 62]. Slower than *x*_k_:*y*_h_ binding rate | s ^-1^ | |
| $\lambda_{x}$ | Degradation of *x* full-length RNA | 4.26×10^-3^ | [58] | s^-1^ | |
| $\lambda_{y}$ | Degradation of *y* full-length RNA | 3.46×10^-3^ | [58] | s^-1^ | |
| $\lambda_{x_{\text{k}}}$ | Degradation of *x*_k_ truncated RNA | 1.42×10^-3^ | [58] | s^-1^ | |
| $\lambda_{y_{\text{h}}}$ | Degradation of *y*_h_ truncated RNA | 1.38×10^-3^ | [58] | s^-1^ | |
| $\lambda_{x:y}$ | Degradation of *x*:*y* hybrid | 1.0×10^-2^ | [62] | s^-1^ | |
| $\lambda_{x_{\text{k}}:y}$ | Degradation of *x*_k_ :*y* hybrid | 1.2×10^-2^ | [62] | s^-1^ | |
| $\lambda_{x:y_{\text{h}}}$ | Degradation of *x*:*y*_h_ hybrid | 1.2×10^-2^ | [62] | s^-1^ | |
| $\lambda_{x_{\text{k}}:y_{\text{h}}}$ | Degradation of *x*_k_:*y*_h_  hybrid | 1.9×10^-2^ | [62] | s^-1^ | |
| k_X_ | X protein translation rate | 3.20×10^-1^ | [67, 68] | nM·(nM·s) ^-1^ | |
| k_Y_ | Y protein translation rate | 5.15×10^-1^ | [67, 68] | nM·(nM·s) ^-1^ | |
| k_XZ_ | Binding rate X:Z | 2.66×10^-1^ | [69] | (nM·s)^-1^ | |
| k_uXZ_ | Unbinding rate X:Z | 85 | [69] | s^-1^ | |
| k_YZ_ | Production of Z via Y protein | 5.9×10^-2^ | Estimated | nM·s ^-1^ | |
| $\lambda_{\text{X}}$ | Degradation of X protein | 1.55×10^-2^ | [69] | s^-1^ |  |
| $\lambda_{\text{Y}}$ | Degradation of Y protein | 1.24×10^-2^ | [69] | s^-1^ | |
| $\lambda_{\text{Z}}$ | Degradation of Z protein | 8.70×10^-3^ | [67] | s^-1^ | |
| $\lambda_{\text{X:Z}}$ | Degradation of X:Z complex | 6.20×10^-3^ | [69] | s^-1^ | |
| K_OY_ | Equilibrium binding constant of X at O_Y_ site | 10.13 | [68, 69] | nM | |
| $\text{μ}$ | Growth rate | 6.0×10^-5^ | [59] | s^-1^ | |
