# Supplementary figures and images for: Cis-Antisense Transcription Gives Rise to Tunable Genetic Switch Behavior: A Mathematical Modeling Approach
Source: PLoS One. 2015 Jul 29;10(7):e0133873. doi: 10.1371/journal.pone.0133873 (PMC4519249; doi:10.1371/journal.pone.0133873)

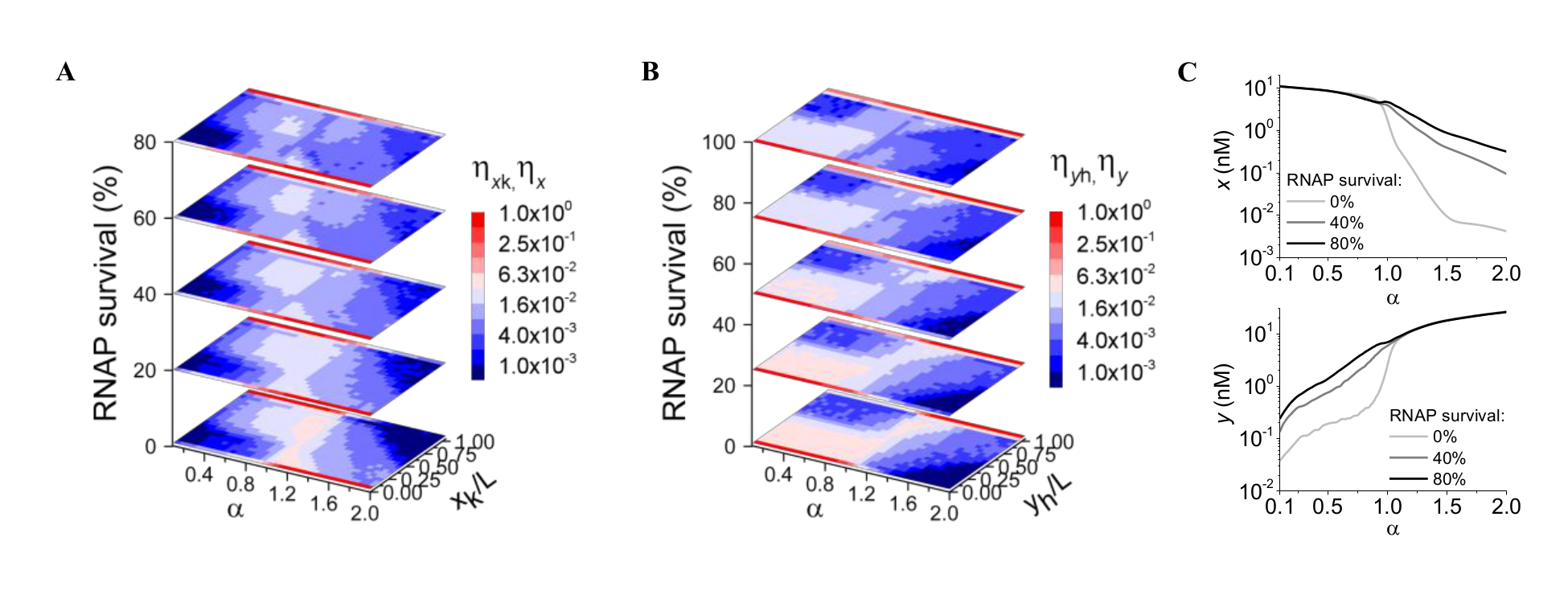

Supplement: S1 Fig — (A, B) Expression maps of full-length (η x, η y) and truncated (ηxk,ηyh) transcripts originating from pX (A) and pY (B), respectively for a range of percentage of RNAP survival in event of RNAP collision. As RNAP survival increases less truncated RNA is produced (bluer central region). (C) Switch response in full-length x and y transcript levels becomes sharper for lower percentage of RNAP survival, at 80% the Hill coefficient is -3.1 for x and 2.9 for y, at 40% it is -4.0 for x and 4.4 for y, and at 0% it is -7.8 for x and 8.2 for y. (TIFF) [file pone.0133873.s002.tiff]

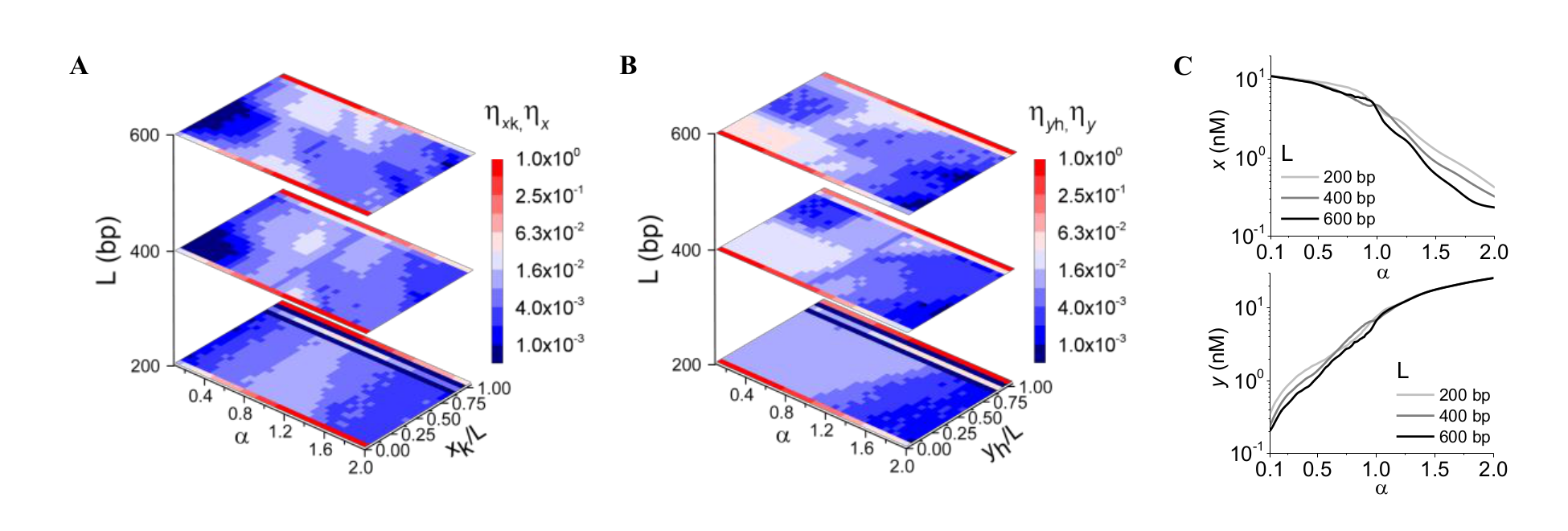

Supplement: S2 Fig — (A, B) Effect of τIX. Maps of truncated and full-length transcripts originating from pX (A) and pY (B), respectively, at different initiation intervals at pX, τIX. Longer initiation times at pX increase probability of sitting duck interference at pX. Dislodgement of more sitting duck complexes at pX cause decreased RNAP collision and lowers expression of full-length x transcripts. (C) The switch response of x and y RNA at different τIX values. x transition becomes sharper at higher τIX values. (D, E) Effect of τIY. As the initiation time at pY increases, analogous effect is seen. Sitting duck interference at pY increases and less RNAP collisions occur. (F) Switch response in x and y levels at different τIY values. y levels are lowered at higher τIY. (TIFF) [file pone.0133873.s003.tiff]

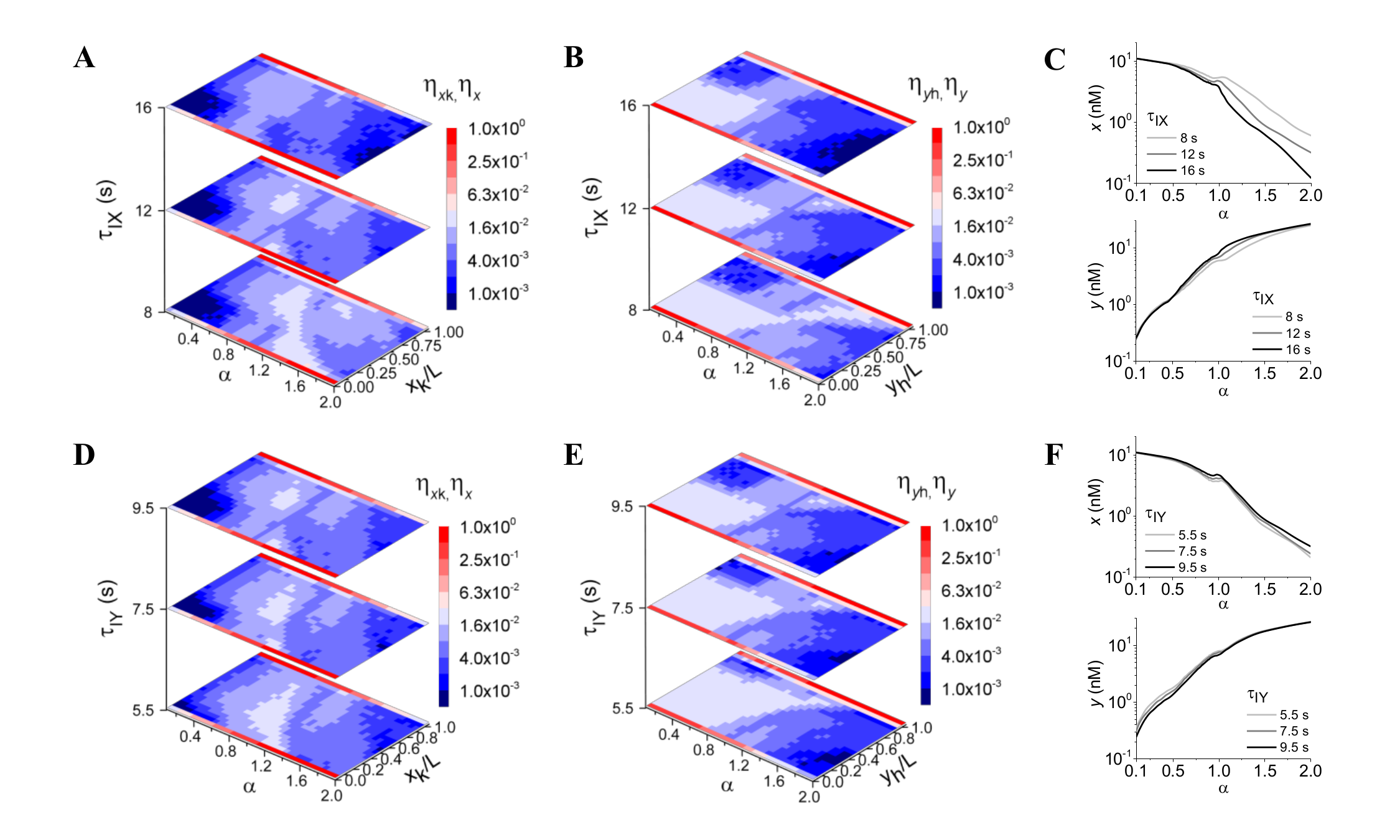

Supplement: S3 Fig — (A, B) Expression maps of truncated and full-length transcripts originating from pX (A) and pY (B), respectively, at different values of overlapping length, L. Longer overlapping regions yield to increased RNAP collisions. (C) Enhanced collisions lower the expression of full-length x and y transcripts. In this case, no clear trend was observed for the Hill coefficient. (TIFF) [file pone.0133873.s004.tiff]

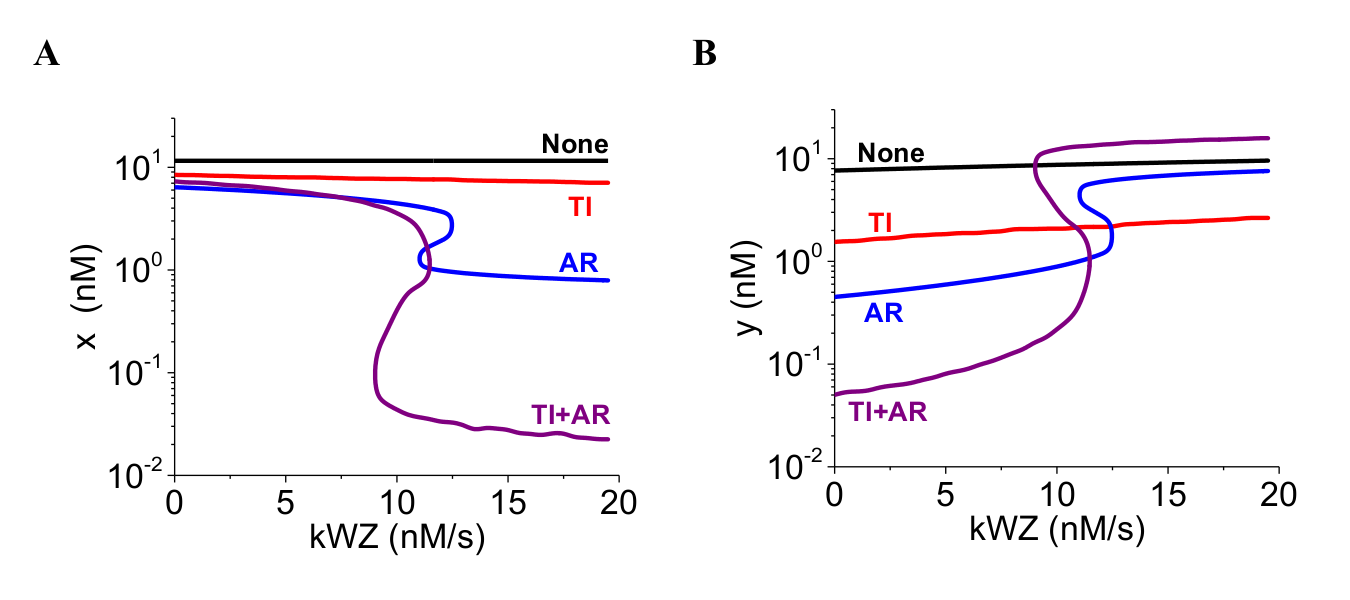

Supplement: S4 Fig — (A, B) Levels of full-length x (A) and y (B) transcripts, respectively, for None, TI, AR and TI+AR cases of antisense transcription in presence of the gene regulatory network implemented by proteins X, Y and Z. Bistability is only observed when AR is present (AR and TI+AR cases). When TI is coupled with AR (TI+AR case) the bistable range is expanded and the switch response is widened. (TIFF) [file pone.0133873.s005.tiff]

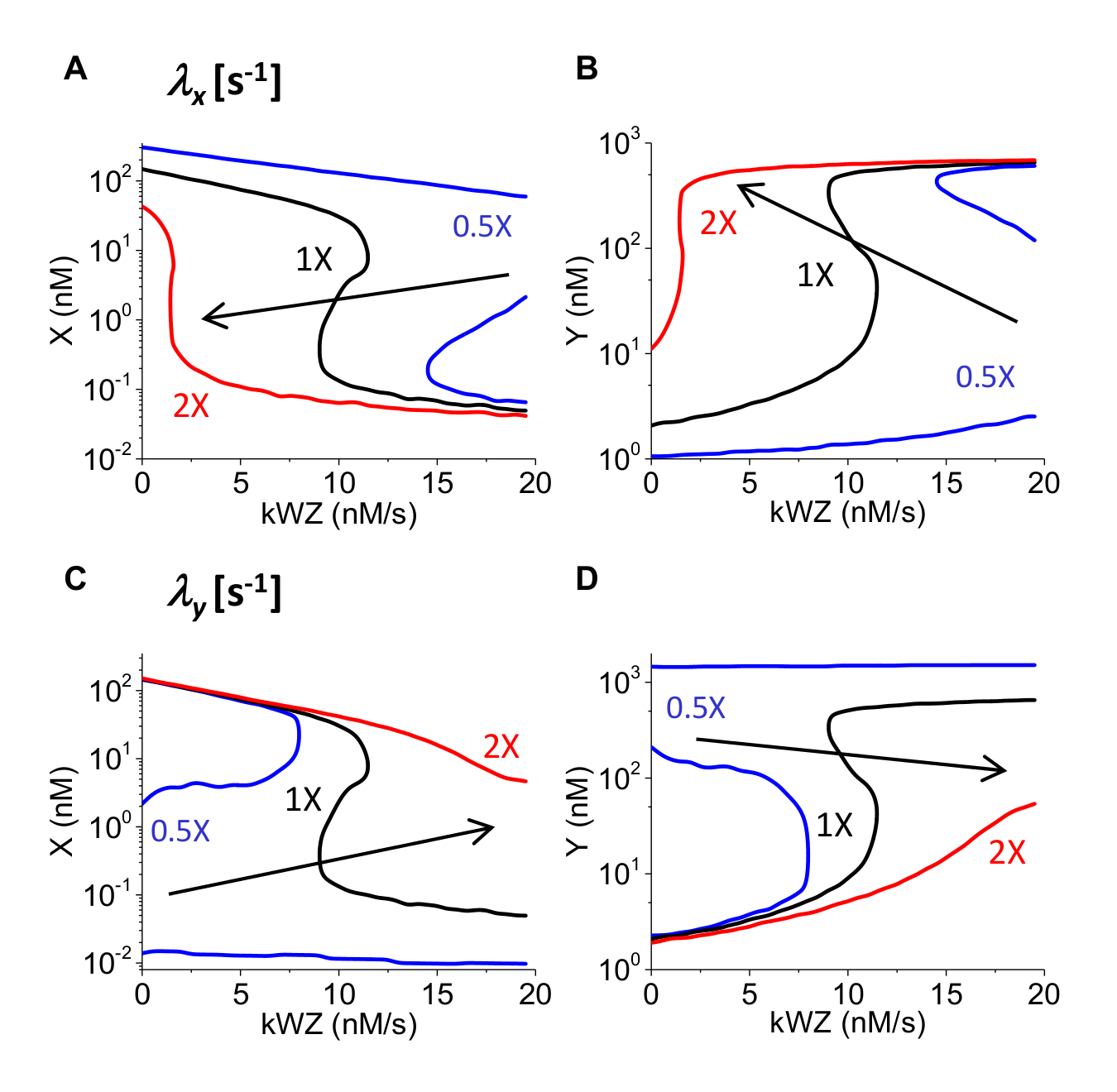

Supplement: S5 Fig — Protein X and Y levels are shown for each modified parameter. Arrows indicate the way the curve shifts as the tuned parameter increases. (A, B) λ x, degradation rate of full-length x. (C, D) λ y, degradation rate of full-length y. (TIFF) [file pone.0133873.s006.tiff]
